# Modified Polycyclic Compounds Rescue Mis-splicing in Myotonic Dystrophy Type 1 Disease Models

**DOI:** 10.1021/acschembio.5c00790

**Published:** 2026-02-20

**Authors:** Jesus A. Frias, Sawyer M. Hicks, Hormoz Mazdiyasni, Subodh K. Mishra, Kahini Sarkar, Clara Yeboah, Noah M. LeFever, Marina M. Scotti, Hana Zeghal, Naomi Brandt, Sweta Vangaveti, Pramita Chakma, Ting Wang, Tammy S. Reid, Omari McMichael, Christopher Crumbaugh, Marina Provenzano, Melissa A. Hale, John D. Cleary, Nicholas E. Johnson, Eric T. Wang, Kaalak Reddy, J. Andrew Berglund

**Affiliations:** † The RNA Institute, College of Arts and Sciences, 1084 University at Albany, State University of New York, Albany, New York 12222, United States; ‡ Department of Biological Sciences, College of Arts and Sciences, University at Albany, State University of New York, Albany, New York 12222, United States; § Department of Molecular Genetics & Microbiology and Center for Neurogenetics, College of Medicine, 12233 University of Florida, Gainesville, Florida 32610, United States; ∥ Department of Chemistry, College of Arts and Sciences, University at Albany, State University of New York, Albany, New York 12222, United States; ⊥ Department of Neurology, 6889 Virginia Commonwealth University, Richmond, Virginia 23284, United States; # Center for Inherited Myology Research, Virginia Commonwealth University, Richmond, Virginia 23298, United States

## Abstract

Myotonic dystrophy type 1 (DM1) is an autosomal dominant multisystemic disorder with no approved therapeutics targeting the disease mechanism. DM1 is caused by the expression of expanded CUG repeat RNA (CUG_exp_), which sequester the muscleblind-like (MBNL) family of RNA binding proteins leading to dysregulated alternative splicing and a host of downstream impacts. While previous studies showed that diamidines rescued DM1 dysregulated alternative splicing events, their potential was limited by toxicity and off-target effects. A new class of modified polycyclic compounds (MPCs), based on diamidines, were created and screened in DM1 patient-derived cell lines. This approach identified MPC03 and MPC04 as being capable of rescuing DM1 dysregulated splicing events at low nanomolar concentrations with no obvious toxicity and limited off-target effects. In a DM1 mouse model, treatment with MPC03 and MPC04 reduced CUG_exp_ RNA levels and partially rescued DM1 mis-splicing. Binding data and modeling showed that lead MPCs bind to CUG_exp_ RNA, and in cells lacking CUG repeats, MPC activity was absent, suggesting that these compounds displace sequestered MBNL proteins from CUG_exp_ RNA. Taken together, MPCs show therapeutic promise across multiple DM1 models.

## Graphical abstract

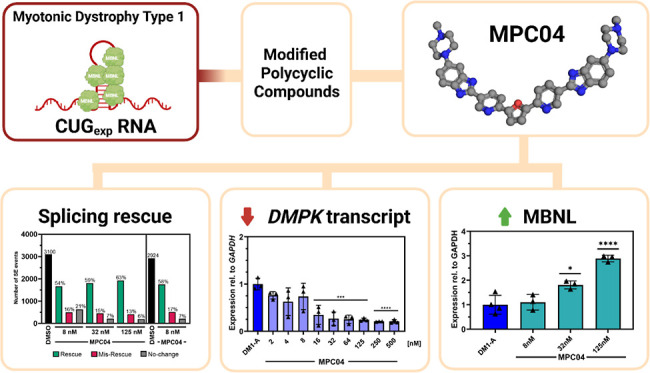

## Introduction

Myotonic dystrophy type 1 (DM1) is the most prevalent form of adult-onset muscular dystrophy,^1^ with a significant personal and financial impact.^2^ DM1 is caused by an unstable CTG repeat expansion in the 3′ untranslated region (3′UTR) of the myotonic dystrophy protein kinase (*DMPK*) gene.^3,4^ The resulting expanded CUG RNA (CUG_exp_) generates a toxic gain-of-function (GOF) effect, whereby CUG_exp_ RNA sequester the muscleblind-like (MBNL) family of RNA binding proteins that regulate alternative splicing.^5,6^ MBNL sequestration causes a loss of regulation in alternative splicing across thousands of splicing events,^7^ many of which have been directly linked to DM1 phenotypes, including myotonia (*CLCN1* exon 7a), cardiac conduction defects (*TNNT2* exon 5), and insulin resistance (*INSR* exon 11).^8–10^ The toxic RNA GOF and further downstream effects of CUG_exp_ expression include repeat-associated non-AUG translation (RAN),^11^ making targeting the RNA an attractive therapeutic option for myotonic dystrophy.

Multiple groups have focused on the therapeutic potential of molecular ligands that bind CUG_exp_ RNA, including both screening existing compounds and designing novel small molecules. These approaches have yielded a variety of small molecules that show binding to CUG repeat RNA and therapeutic efficacy, including aminoglycosides,^12^ Hoechst derivatives,^13^ and various single, dimeric, or multivalent ligands.^14–18^ Our group has previously focused on diamidines and reported several compounds that can rescue DM1-associated dysregulated splicing in patient-derived cells and the DM1 *HSA* ^LR^ mouse model, including pentamidine, heptamidine, and furamidine.^19–23^ Several of these compounds, including pentamidine and furamidine, have been previously tested as antiparasitics in clinical trials but were halted due to liver and kidney toxicity.^24^ Testing in DM1 models showed that these compounds rescued DM1 dysregulated splicing events and reduced levels of CUG_exp_ RNA;^20,22,25^ however, rescue occurred at or near toxic concentrations. Small-scale screening with furamidine and various modified diamidines, including DB1247 and compound 7,^26^ also demonstrated rescue of dysregulated splicing events across multiple DM1 fibroblast cell lines. The lead compound from that study showed an improved efficacy–toxicity profile relative to furamidine, suggesting that further modifications of structurally similar small molecules could yield an improved therapeutic candidate.

In this work, we designed, synthesized, and tested a series of small molecules named modified polycyclic compounds (MPCs), inspired by diamidine compounds. These MPCs are composed of three basic elements: a heterocyclic core, benzimidazole side groups, and functionalized end groups. Here, we screened a limited set of MPCs in a DM1 patient-derived fibroblast cell line and identified modifications that showed rescue of dysregulated splicing events in the low nanomolar (defined here as <100 nM) concentration range. Further testing in DM1 patient-derived myotube cell lines validated two lead compounds, MPC03 and MPC04, as being able to rescue splicing, reduce *DMPK* expression, and increase muscleblind-like 1 (MBNL1) protein expression at nanomolar concentrations. These lead compounds showed no cellular toxicity up to a 1000-fold higher concentration than required for splicing rescue. Testing of lead MPCs in the *HSA* ^LR^ DM1 mouse model demonstrated modest but significant effects on splicing rescue and reduction in CUG transgene expression. These results support MPCs as compounds capable of disrupting DM1 disease pathology, rescuing dysregulated splicing, gene expression, and protein expression with low to no cellular toxicity.

## Results and Discussion

### MPCs Rescue Mis-splicing in a DM1 Patient-Derived Fibroblast Cell Line

We have previously shown that diamidine compounds, including furamidine, rescue DM1 mis-splicing but with noted off-target effects and toxicity.^26^ Using these diamidines as an inspiration, we developed a series of modified polycyclic compounds (MPCs) that build off three core elements: a heterocyclic core, benzimidazole side groups, and functionalized end groups (Figure 1A). Ten different MPC compounds (Figure 1B,C) were synthesized, analyzed for their druglikeness (Table S1), and tested in a previously characterized DM1 patient-derived fibroblast cell line (GM04601).^26^ The inclusion levels of cassette exons for two different alternative splicing events (*INSR* exon 11 and *FLNB* exon 31) previously shown to be mis-spliced in DM1 cell models^26,27^ were used to assess MPC activity (Table 1). Fibroblasts were treated with each compound for 96 h, and RNA was extracted. RT-PCR analysis was performed to calculate percent spliced in (PSI) (eq 1) and splicing percent rescue (% rescue) (eq 2):

1 $$PSI=\left[\frac{inclusion}{\left(inclusion+exclusion\right)}\right]\times 100\%$$

2 $$\%\;rescue=\left[\frac{\left(affected\;PSI-treated\;PSI\right)}{\left(affected\;PSI-unaffected\;PSI\right)}\right]\times 100\%$$

**Figure 1:**
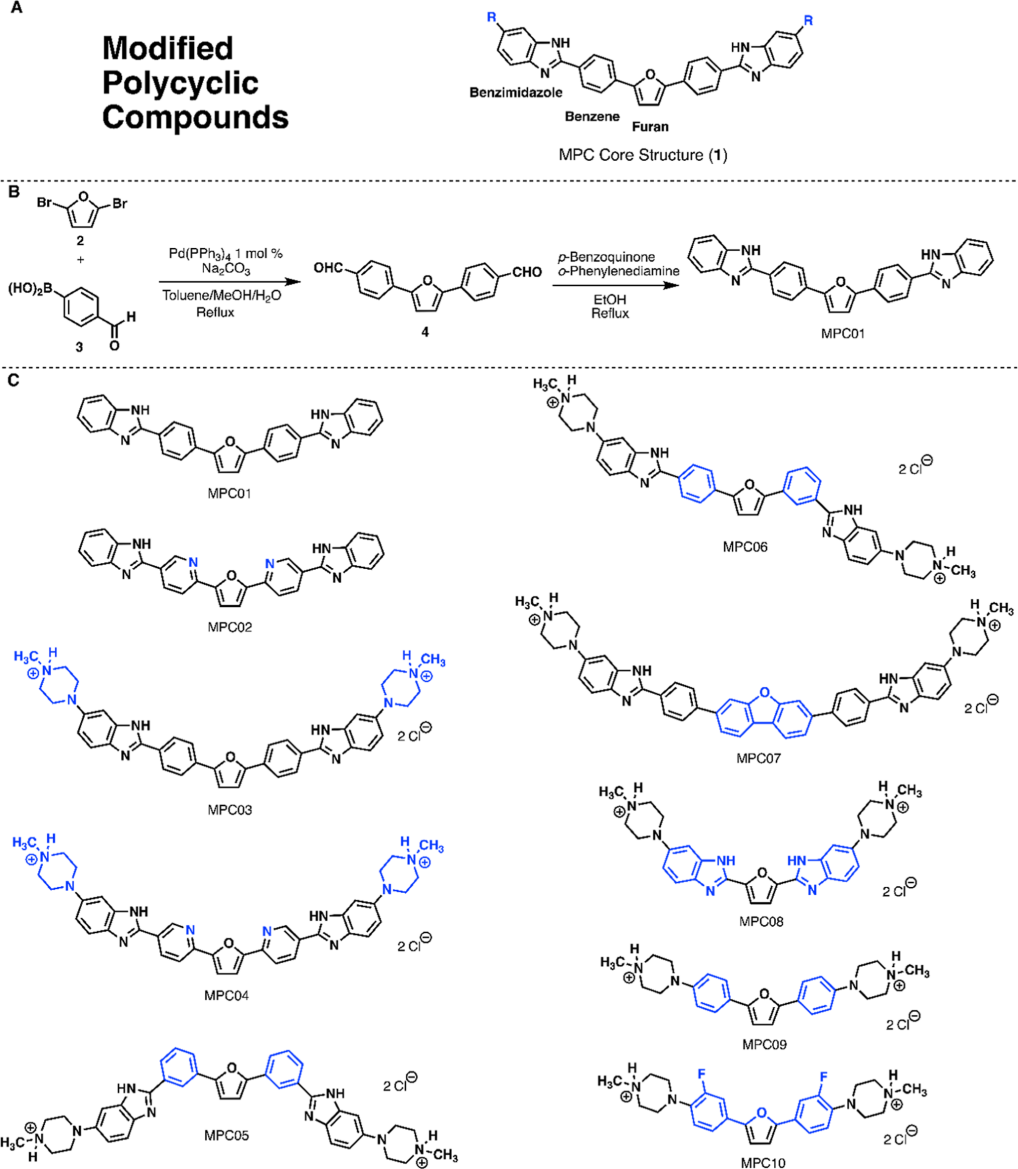
Structures of modified polycyclic compounds (MPCs). (A) Core structure of our MPC molecules containing furan, benzene/pyridine, and benzimidazole motifs. (B) Synthetic route of MPC01; (C) changes to the core structure produces MPC01–10. Placing a H as the R end group in the core structure leads to MPC01, while changing the benzene rings to pyridines leads to MPC02. Replacing the R end group with an *N*-methyl piperazine in MPC01 and MPC02 leads to MPC03 and MPC04, respectively. Changing from a para to meta for the side and end groups on both sides leads to MPC05, while opposite para and meta orientations to MPC06. Substitution of a central furan core with a dibenzofuran leads to MPC07, while removal of side-group benzene rings adjacent to the central furan core leads to MPC08. Removal of benzimidazole side groups leads to MPC09, and replacing benzenes with fluorobenzene rings leads to MPC10.

**Table 1:** MPC Splicing Metrics for *INSR* Exon 11 and *FLNB* Exon 31^a^

	*FLNB* exon 31			*INSR* exon 11		
MPC	EC_50_	EC_max_	*p*-adj	EC_50_	EC_max_	*p*-adj
MPC01	62.2	21.6	yes	86.3	20.5	yes
MPC02	3.67	11.6	yes	24.1	8.25	yes
MPC03	3.81	23.2	yes	4.21	24.8	yes
MPC04	3.5	32.7	yes	3.49	23.4	yes
MPC05	–	4.9	no	–	6.85	no
MPC06	497	6.3	yes	–	4.12	no
MPC07	–	1.7	no	–	1.39	no
MPC08	–	2.9	no	–	2.17	no
MPC09	–	2.68	no	528	8.4	yes
MPC10	–	1.95	no	–	4.85	no

a EC50: concentration (nM) needed to reach 50% of effect; EC Max: maximum ΔPSI reached; *p*-adjusted (*p*-adj) values obtained from a one-way ANOVA with multiple comparisons to the untreated DM1 fibroblasts (yes≤ 0.05, no > 0.05); spaces with “-” indicate that EC50 was not calculated if ΔPSI was not significantly changed from untreated DM1 fibroblasts.

To explore different MPC modifications, we first focused on modifications to the benzimidazole side groups and functionalized end groups. Treatment with MPC01 rescued *INSR* exon 11 and *FLNB* exon 31 mis-splicing beginning at 64 nM and showed maximal rescue at 1 μM of 75.3 ± 11.5% and 64.9 ± 3.8%, respectively (Figure 2A). The benzene rings of MPC01 were changed to pyrimidine groups to generate MPC02 (Figure 1C). Treatment with MPC02 did not significantly rescue *INSR* exon 11 mis-splicing but it did rescue *FLNB* exon 31 beginning at 32 nM and showed a maximal rescue of 39 ± 6.9% at 1 μM (Figure 2B). To explore the role of the functionalized end groups, we added methylpiperazine groups to either side of the 4-position carbon of the benzimidazole groups of MPC01 to generate MPC03 (Figure 1C). Treatment with MPC03 showed a notable improvement in splicing rescue (Figure 2C) with an optimal splicing rescue of 81 ± 6.9% at 32 nM for *INSR* exon 11 and 62 ± 3.3% at 64 nM for *FLNB* exon 31. A similar functionalized end group modification on the pyrimidine ring-based MPC02 generated MPC04 (Figure 1C). Treatment with MPC04 showed greater splicing rescue at lower concentrations than MPC03 (Figure 2D) with a 72 ± 9.9% rescue of *INSR* exon 11 and a 54 ± 8.2% rescue of *FLNB* exon 31 at 16 nM. Importantly, none of the MPCs showed cellular toxicity at the low nanomolar concentrations, which rescue dysregulated splicing and only started to show toxicity at 1000× concentration (32–64 μM for MPC03 & 04) (Figure S1). Our results showed that modifications to the end group in MPC03 and MPC04 resulted in significant improvements, with MPC04 achieving the highest splicing rescue at lower concentrations than previously tested compounds.^20,22,26^

**Figure 2:**
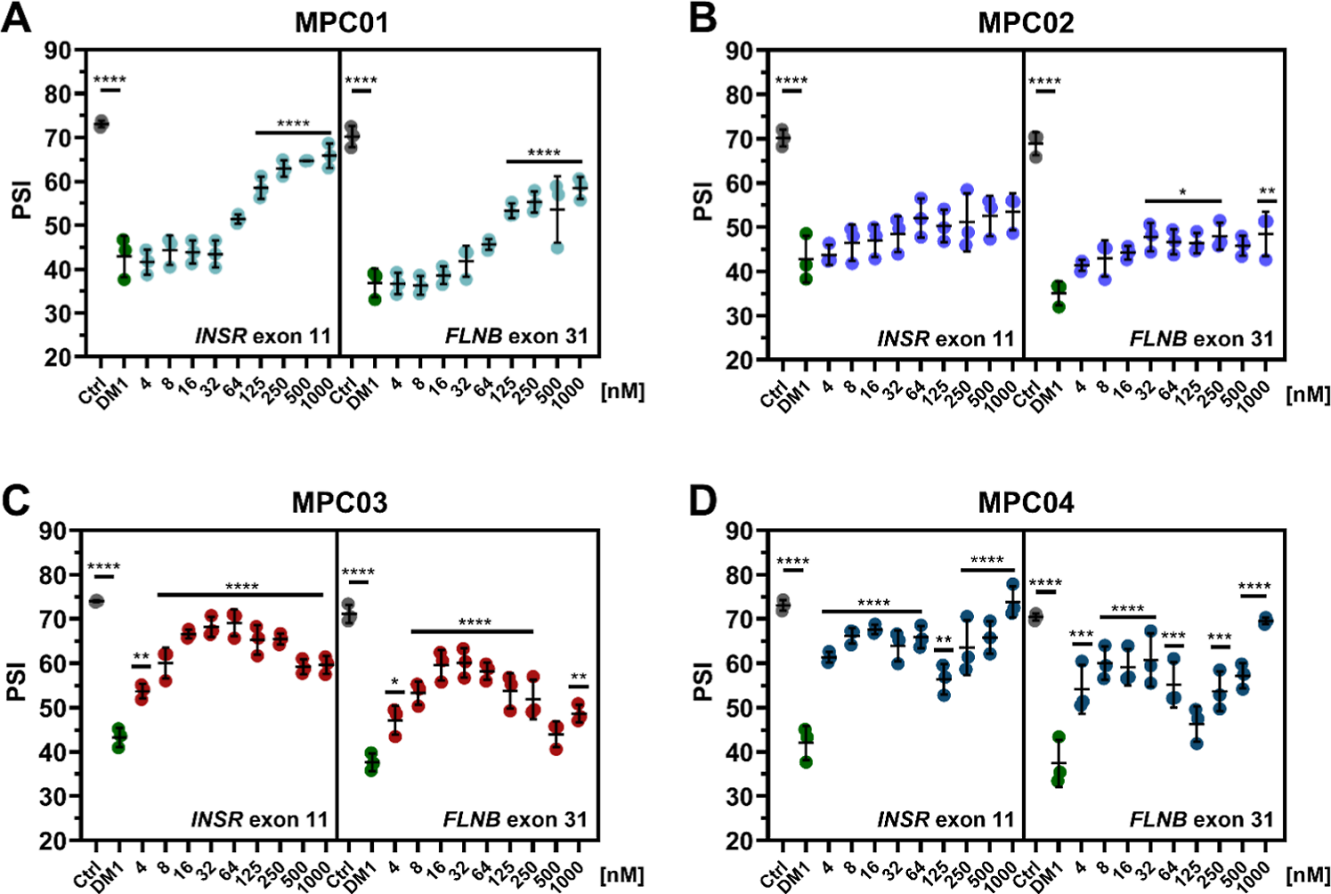
MPCs partially rescue dysregulated splicing at nanomolar concentrations in DM1 patient-derived fibroblasts. RT-PCR isoform analysis of cassette exons (*INSR* exon 11 and *FLNB* exon 31) previously shown to be dysregulated in DM1 was carried out to determine inclusion levels after 96 h of treatment with MPCs. Percent spliced in (PSI) between control and DM1 fibroblasts treated with DMSO or MPC01 (A), MPC02 (B), MPC03 (C), and MPC04 (D) was used to assess mis-splicing rescue. Mean ± standard error of the mean, Ctrl = unaffected individual fibroblasts (*n* = 24), 0 = DMSO-treated DM1 fibroblasts (*n* = 32), MPC-treated DM1 cells had *n* = 3 for each concentration. One-way ANOVA with Dunnet’s multiple comparisons to DMSO-treated fibroblasts (*P*-adj <0.05 = *, <0.01**, <0.0001***, ns = nonsignificant).

Next, we explored how alterations to the MPC core affected the splicing rescue. The attachment sites of benzimidazole groups of MPC03 were changed to the 3-position carbons of either benzene ring, from a para position to meta, resulting in MPC05 (Figure 1C). The change in orientation of the benzimidazole and methylpiperazine groups eliminated the ability to rescue splicing of *INSR* exon 11 and *FLNB* exon 31 (Figure S2A). Moving the orientation of only one side group of MPC03 from para to meta resulted in MPC06 (Figure 1C), which also eliminated splicing rescue (Figure S2B). Additionally, substitution of the furan core ring of MPC03 with dibenzofuran (MPC07, Figure 1C) or removal of the benzene rings of MPC03 (MPC08, Figure 1C) eliminated splicing rescue (Figure S2C,D). Further modifications to the MPC core, including removing the benzimidazole groups (MPC09, Figure 1C) or substitution of fluorobenzene rings (MPC10, Figure 1C) also eliminated splicing rescue (Figure S2E,F). Taken together, these results demonstrate that the MPC core groups and their orientation to each other are all critical components for splicing rescue activity.

### MPCs Rescue Mis-splicing and Affect *DMPK* and *MBNL1* Expression in Two DM1 Myotube Models

To further explore the potential of the three most effective MPCs (MPC02, MPC03, and MPC04), we evaluated effects on splicing in DM1 (DM1-A) patient-derived myotubes from an individual containing ∼2900 CTG repeats.^28^ Myoblasts were differentiated for 96 h into myotubes as previously described,^20^ treated with a concentration range of MPCs (2 nM to 1 μM) for 96 h, and then tested for splicing rescue (Figure 3A,C,E) and cell viability (Figure 3B,D,F). For splicing rescue, we used two well-known dysregulated splicing events in DM1 myotubes, *MBNL1* exon 5 and *NUMA1* exon 16.^20,22^ All three lead MPCs partially rescued dysregulated splicing for these events. MPC02 showed a modest but not statistically significant 19% rescue at 1 μM for *MBNL1* exon 5 and showed a stronger significant rescue of 24% at 500 nM for *NUMA1* exon 16 (Figure 3A). MPC03 significantly rescued both events to a greater degree than MPC02, with 35% rescue at 1 μM for *MBNL1* exon 5 and 39% rescue at 1 μM for *NUMA1* exon 16 (Figure 3C). MPC04 demonstrated the greatest splicing rescue at 125 nM with 71% for *MBNL1* exon 5 and at 62.5 nM with 58% for *NUMA1* exon 16 (Figure 3E). MPC04 outperformed the previously tested diamidine, furamidine, which required a 20-fold higher concentration for a maximum rescue of 30% and 22% for these events, respectively.^22^ MPC02 and MPC03 treatments did not show toxicity until 1 μM and 10 μM, respectively (Figure 3B,D). MPC04 exhibited no toxicity in our testing up to 10 μM, which is an 80-fold difference from the concentration that demonstrated optimal splicing rescue (Figure 3F). Interestingly, there was a modest increase in cell viability observed for MPC04 treatment at intermediate values (Figure 3F), which may reflect reduced stress following a reduction in the level of toxic RNA effects.

**Figure 3:**
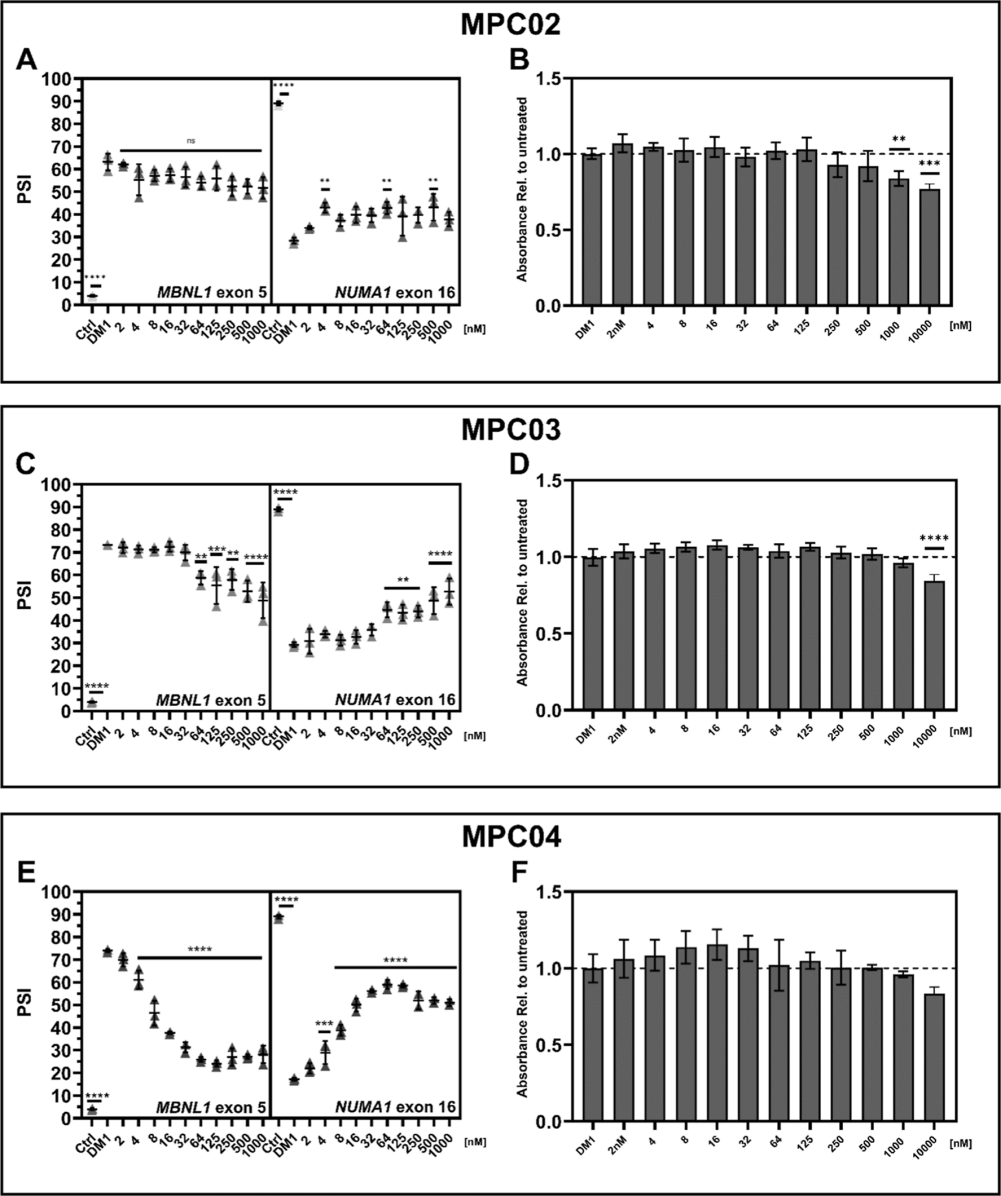
Lead MPCs partially rescue mis-splicing in patient-derived myotubes without affecting cell viability. RT-PCR isoform analysis of *MBNL1* exon 5 and *NUMA1* exon 16 spliced in levels in DM1-A cell line demonstrates DM1-mis-splicing rescue in myotubes with MPC treatment. (A) MPC02, (C) MPC03, and (E) MPC04 all significantly rescued both events. HS Presto blue assay with DMSO- and MPC-treated DM1 myotubes shows that (B) MPC02, (D) MPC03, and (F) MPC04 do not decrease myotube viability within the concentration range at which mis-splicing rescue is observed. Adjusted-*P* values were determined with one-way ANOVA and Dunnett’s multiple comparisons to the untreated DM1 values (*n* = 3 for RT-PCR analysis, *n* = 4 for HS Presto blue assay) (*P*-adj <0.05 = *, <0.01**, <0.0001***, ns = nonsignificant).

To investigate the activity of MPC04 in an additional patient-derived DM1 myotube cell model, we selected a line from a female patient carrying ∼1300 CTG repeats (DM1-B).^29^ Characterization of DM1-B cells revealed less severe splicing dysregulation for *MBNL1* exon 5 and *NUMA1* exon 16 than DM1-A, and RNA-seq analysis of DM1-B showed 1344 shared splicing events with a slightly higher mean absolute delta PSI (34 ± 16) than DM1-A (31 ± 16) (Figure S3). Following treatment of DM1-B with MPC04, we observed splicing rescue at low nM concentrations, with a significant rescue of *MBNL1* exon 5 (81 ± 3% rescue at 2 nM, *p*-adj <0.05) and *NUMA1* exon 16 (60 ± 1.8% rescue, at 4 nM, *p*-adj <0.05) (Figure 4A,B). Comparing the dose–response curve between the two DM1 myotube cell lines, the EC50 for DM1-B was lower (1.6 nM for *MBNL1* exon 5 and 1.5 nM for *NUMA1* exon 16) than DM1-A (6.3 nM for *MBNL1* exon 5 and 6.3 nM for *NUMA1* exon 16). These experiments demonstrated that lower MPC04 concentrations were required to rescue splicing in the DM1-B line, which contains fewer repeats than DM1-A.

**Figure 4:**
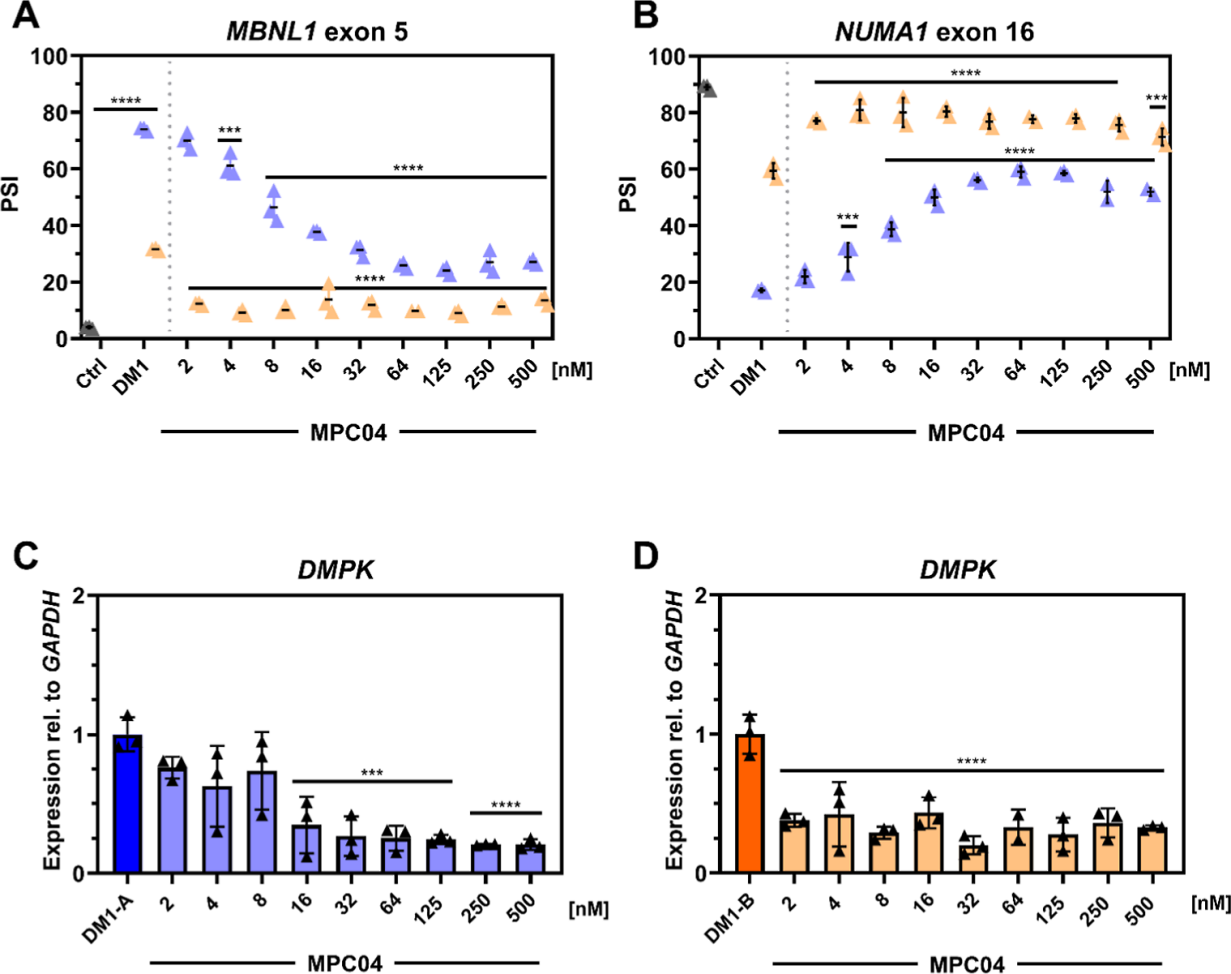
MPC04 rescues mis-splicing in multiple DM1 myotube cell lines and reduces *DMPK* transcript levels. DM1-A myotubes (2900 CTG repeats, blue data points) show greater mis-splicing of (A) *MBNL1* exon 5 and (B) *NUMA1* exon 16 compared to DM1-B myotubes (∼1317 CTG repeats, orange data points), determined by RT-PCR isoform analysis. MPC04 partially rescued mis-splicing of *MBNL1* exon 5 and *NUMA* exon 16 at lower concentrations in DM1-B than in DM1-A myotubes. Relative *DMPK* transcript levels (normalized to *GAPDH*) were reduced in (C) DM1-A and (D) DM1-B myotubes, determined by RT-qPCR. Adjusted-*P* values were determined with one-way ANOVA and Dunnett’s multiple comparisons to the untreated DM1 values (*n* = 3 for all treatments, mean ± SEM) (*P*-adj <0.05 = *, <0.01**, <0.0005***, <0.0001****).

To better understand the effects of MPC04 on other aspects of CTG repeat pathogenesis in DM1, we performed qPCR to determine the expression levels of *DMPK* in DM1-A and DM1-B myotubes treated with MPC04. We found that MPC04 treatment reduced *DMPK* expression at concentrations where splicing rescue was observed in both DM1-A and DM1-B myotube models (Figure 4C,D). We confirmed that the reduction in *DMPK* levels following MPC04 treatment coincided with a reduction in CUG ribonuclear foci (Figure S4). We next measured the expression of *MBNL1* and *MBNL2* RNA and observed an increase in the expression of both transcripts in both DM1-A and DM1-B myotube models (Figure S5A–D). In the DM1-A myotubes, we saw a dose–response in *MBNL1* expression that matched the *MBNL1* exon 5 and *NUMA1* exon 16 splicing, reaching a 2-fold change in expression at 125 nM (Figure S5A). In DM1-B myotubes, we observed a near 2-fold change in *MBNL1* transcript expression beginning at 2 nM (Figure S5B). To confirm our observation of elevated *MBNL1* RNA expression, we measured MBNL1 protein levels and found elevated levels in both DM1 myotube models after MPC04 treatment (Figure S6A–D). *MBNL2* RNA expression was consistently elevated at most treatment concentrations (Figure S5C,D). Taken together, these data support an increase in both MBNL transcript and protein expression following MPC treatment.

### MPC04 Rescued Hundreds of Skipped-Exon Events in DM1 Myotubes with Little Off-Target Effects

To further investigate the extent of splicing rescue by MPC04, we performed RNA-seq on Ctrl, DM1-A, and DM1-B myotubes treated with either 0.1% DMSO (v/v, vehicle control) or different concentrations of MPC04. We identified 3100 dysregulated skipped-exon (SE) events in DM1-A myotubes and 2924 in DM1-B myotubes. Analysis of RNA-seq data for *MBNL1* exon 5 and *NUMA1* exon 16 splicing showed rescue similar to RT-PCR analysis (Figure 4A,B vs [Fig fig5]A,B), along with rescue of many other DM1 associated mis-splicing events (Figure 5D,E). To determine global splicing rescue, we used percent rescue score (eq 2) and categorized all events as either “Rescue” (score ≥10%), “Mis-Rescue” (score ≤ −10%), or “No-change” (score < −10% and <10%). In both DM1-A and DM1-B myotubes, we observed a greater number of “Rescue” events than “Mis-Rescue” or “No-change” (Figure 5D). The number of rescued events increased in a dose-dependent manner in DM1-A (1663 events at 8 nM, 1804 events at 32 nM, and 1924 events at 125 nM). Similarly, “Mis-Rescue” events decreased in a dose-dependent manner in DM1-A myotubes (495 events at 8 nM, 453 events at 32 nM, and 411 events at 125 nM). The low treatment concentration in DM1-B myotubes had a slightly higher number of rescued events compared to the same treatment concentration in the DM1-A myotubes. MPC04 partially rescued approximately 60% of the dysregulated skipped exon events in two DM1 patient-derived cell lines. The remaining events that are not rescued may require higher levels of available MBNL proteins to regulate splicing.

**Figure 5:**
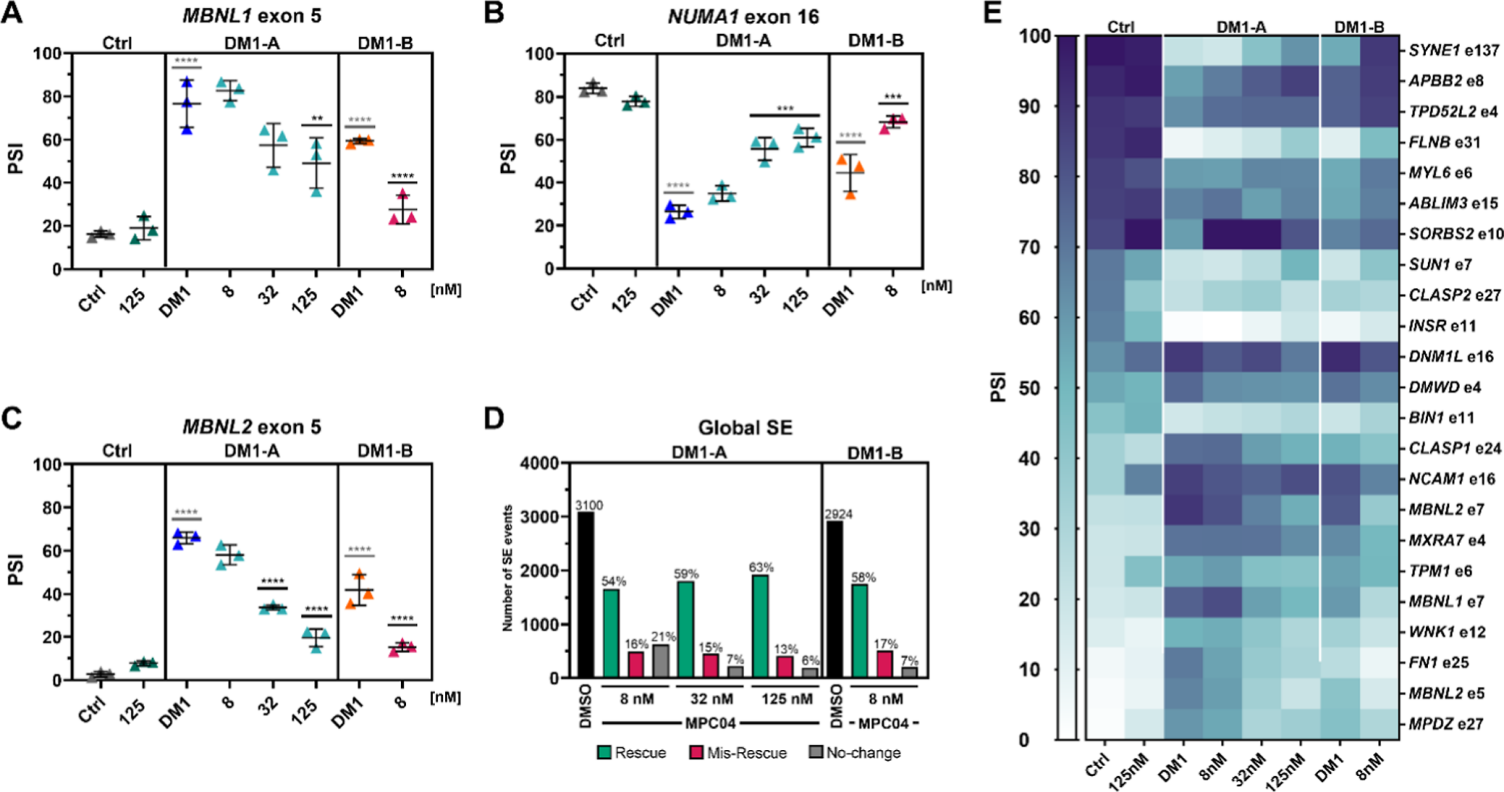
MPC04 rescues thousands of SE events in DM1 myotubes. (A) *MBNL1* exon 5, (B) *NUMA1* exon 16, (C) and *MBNL2* exon 5 events response to treatment by MPC04 in DM1-A and DM1-B myotubes after 96 h of treatment. (D) Total number of skipped-exon (SE) events dysregulated in DM1-A (left, black bar) and DM1-B (right, black bar), and the effect of MPC04 treatment at different concentrations (8, 32, and 125 nM). After treatment, dysregulated events were categorized as either rescue (green), mis-rescue (red), and no-change (gray). The percentage of SE events for each group with respect to the dysregulated total (black bar) within each cell line is displayed above (*P*-adj <0.05 = *, <0.01**, <0.0005***, <0.0001****). (E) Heatmap of 23 dysregulated skipped exon splicing events after MPC04 treatment in control, DM1-A, and DM1-B cell lines.

To understand how MPC04 affects splicing in the absence of expanded repeats, Ctrl cells were treated with either MPC04 at 125 nM or vehicle control. Of the 1058 observed SE events changed by MPC04 treatment, only 96 events had a difference in PSI >30% (Figure S7A) with 171 overlapping SE events with the two DM1 cell lines (Figure S7B). These findings demonstrate that MPC04 is capable of rescuing thousands of dysregulated splicing events found in DM1 with relatively minimal off-target activity.

We next assessed the global effect on gene expression from MPC04 treatment using a similar percent rescue score (eq 3):

3 $$\%\;rescue=100\%\times \left[1-\frac{\left(unaff.\;\mathrm{expr}.-aff.\;\mathrm{expr}.\right)+\left(treated\;\mathrm{expr}.-aff.\;\mathrm{expr}.\right)}{\left(unaff.\;\mathrm{expr}.-aff.\;\mathrm{expr}.\right)}\right]$$

where “aff.”, “unaff.”, and “expr.” denote “affected”, “unaffected”, and “expression”, respectively. The % rescue scores were used for categorization (rescue, mis-rescue, and no-change). Of the 2551 dysregulated genes in DM1-A myotubes and 2601 dysregulated genes in DM1-B myotubes, more events were categorized as “Rescue” than “Mis-Rescue” or “No-change” (Figure S8A). Across the three concentrations used to treat DM1-A myotubes, we observed a dose-dependent effect with the percentage of “rescue” dysregulated genes increasing (gene counts are 1042 at 8 nM to 1281 at 32 nM to 1460 at 125 nM), “No-change” decreases (gene counts are 985 at 8 nM to 705 at 32 nM to 524 at 125 nM), while “Mis-Rescue” genes remained constant. The DM1-B myotubes exhibited an effect at 8 nM similar to that of the DM1-A myotubes. We performed a gene-ontology biological process analysis using Enrichr^30–32^ of the shared “Rescue” genes from 125 nM MPC04 treatment DM1-A myotubes and at 8 nM treated DM1-B myotubes. Some of the most significantly enriched terms for “Rescued” genes (Figure S8B) included muscle processes including muscle organ development (GO:0007517) and skeletal muscle contraction (GO:0003009). Taken together, these data demonstrate that similar to splicing, MPC04 is capable of rescuing thousands of dysregulated gene expression events with relatively limited off-target gene expression activity.

### MPC04 Is Capable of Modulating Splicing and Gene Expression in the HSA^LR^ Mouse Model

To further evaluate the potential of MPCs as small molecule therapeutics for DM1, we next tested our two lead candidates, MPC03 and MPC04, in the widely used human skeletal actin long repeat (HSA^LR^) mouse model.^33^ These mice express ∼220 CTG repeats from the human skeletal actin (*ACTA1*) promoter, resulting in myotonia, muscle weakness, and reduced muscle fiber size. Small cohorts (*n* = 4) of HSA^LR^ and wild-type (WT) mice were treated by intraperitoneal (IP) injections every day for 5 days with either 20 mg/kg of MPC03, MPC04, or a DMSO/PBS solution (1:1 v/v). Tibialis anterior muscle was collected 24 h after the fifth injection, and RNA was extracted. RT-PCR splicing analysis of this RNA was conducted for three events commonly reported from HSA^LR^ studies, *Atp2a1* exon 22, *Clcn1* exon 7a, and *Mbnl1* exon 5. We observed greater rescue for MPC03 than MPC04 across *Atp2a1* exon 22 (65% vs 33%), *Mbnl1* exon 5 (68% vs 23%), and *Clcn1* exon 7a (76% vs 49%) (Figure 6A–C). Interestingly, the greater rescue by MPC03 over MPC04 contrasts the results we observed in patient-derived myotubes (Figure 3). Additionally, we observed a significant decrease in *ACTA1* transgene levels in mice following treatment with MPC03 (80%) and MPC04 (30%) (Figure 6D). Interestingly, no changes in endogenous *Dmpk* or *Mbnl1* expression levels were found after treatment by either MPC (Figure 6E,F). The reduction of the *ACTA1* transgene and lack of effect for endogenous *Dmpk* or *Mbnl1* support the therapeutic potential of our lead MPCs and suggest in this system that their in vivo activity acts through a reduction in CUG_exp_ RNA levels.^34^

**Figure 6:**
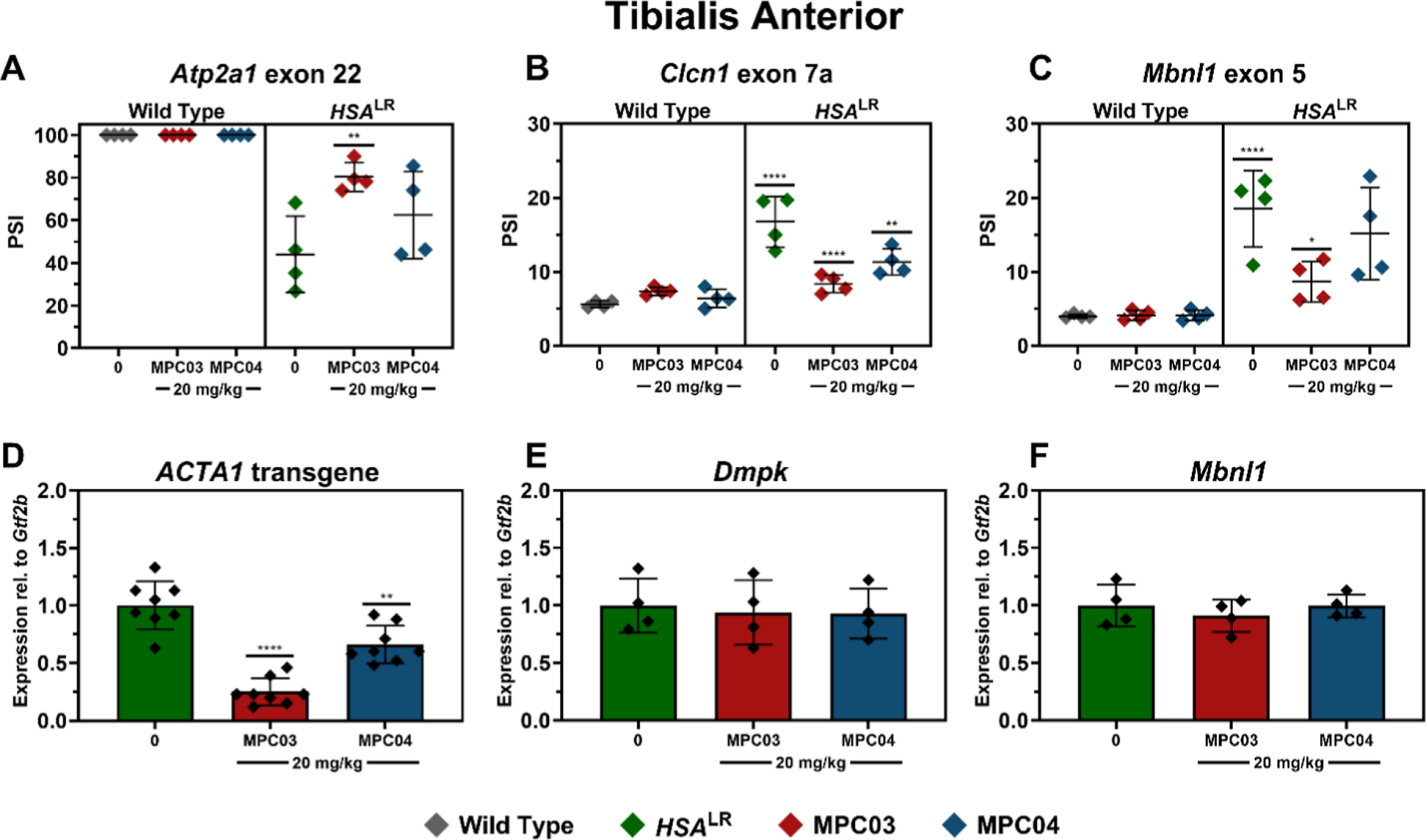
MPCs partially rescue mis-splicing and reduce CUG RNA levels in the *HSA* ^LR^ DM1 mouse model. RT-PCR isoform analysis of RNA from tibialis anterior muscle from wildtype (WT) or *HSA* ^LR^ DM1 mice treated with vehicle (PBS-DMSO) or 20 mg/kg of MPC03 or MPC04. (A) *Atp2a1* exon 22 was partially rescued by MPC03, while both (B) *Clcn1* exon 7a and (C) *Mbnl1* exon 5 were partially rescued by both MPCs. RT-qPCR transcript level analysis showed a reduction of (D) relative ACTA1 transgene levels (which contain repeat CUG RNA) and no effect on endogenous (E) *Dmpk* and (F) *Mbnl1* levels. Adjusted-*P* values were determined with one-way ANOVA and Dunnett’s multiple comparisons to the untreated DM1 values (*n* = 4 for all treatments) (*P*-adj <0.05 = *, <0.01**, <0.0005***, <0.0001****).

### MPC04 Does Not Affect Splicing and Gene Expression in C2C12 Mouse Myoblasts

To further understand the relationship between *Dmpk* and CUG_exp_ RNA expression, we used C2C12 mouse myoblasts to examine splicing and gene expression outside the context of a CUG_exp_ RNA. C2C12 myoblasts contain only 1 CTG in the 3′UTR of *Dmpk*, unlike typical unaffected human myoblasts, which contain between 5 and 34 CTG repeats in the 3′UTR of *DMPK*.^35^ Following a 96 h treatment with 125 nM MPC04, RNA was extracted from C2C12 mouse myoblast cells and splicing and gene expression were measured. We found that neither *Dmpk* nor *Mbnl1* expression levels changed after treatment and as expected splicing of *Mbnl1* exon 5 and *Atp2a1* exon 22 showed no significant changes (Figure S9). We then measured *DMPK* and *MBNL1* expressions in human Ctrl2 myoblasts treated with MPC04 for 96 h. We observed a significant reduction in *DMPK* expression at 2 nM, indicating that MPC04 activity is related to CUG RNA expression (Figure S10A). We also observed a trend toward a dose-dependent increase in *MBNL1* RNA expression, which was mirrored by MBNL1 protein expression (Figure S10B–D). Taken together, these data suggest that our lead MPCs operate through binding or interacting with CUG_exp_ RNA with feedback on *MBNL* transcript levels, with splicing rescue appearing to be closely linked to the reduction of CUG_exp_ RNA levels.

### MPC04 Binds CUG RNA

To evaluate the binding specificity of MPC04 toward CUG repeat RNA, isothermal titration calorimetry (ITC) experiments were performed using r­(CUG)_8_ RNA. A palindromic DNA duplex, d­(CTG)_4_(CAG)_4_, was used as a control to assess the selectivity. The ITC data demonstrated strong binding between MPC04 and r­(CUG)_8_ RNA (Figure 7A). The binding isotherm was best fit using a multisite binding model, yielding two dissociation constants, *K*_d1_ = 1 ± 0.3 nM and *K*_d2_ = 3 ± 2 nM, indicative of high-affinity interactions. The corresponding binding stoichiometries (*N*_1_ = 9 ± 2, *N*_2_ = 8 ± 2) suggest that multiple MPC04 molecules bind to a single r­(CUG)_8_ RNA duplex, consistent with the presence of repeated binding sites along the RNA structure.

**Figure 7:**
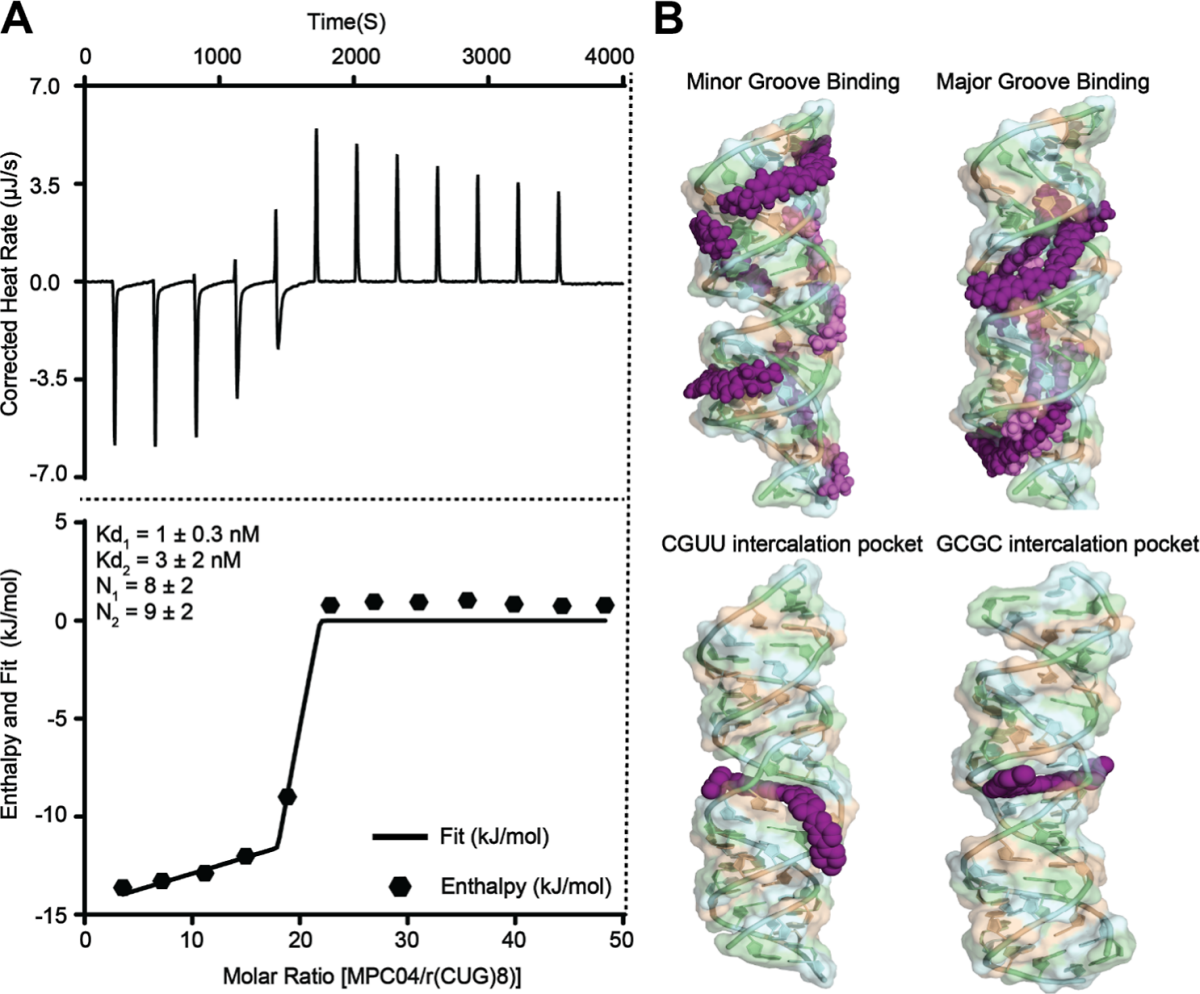
ITC-based binding affinity and molecular docking-based interaction model of MPC04 with r­(CUG)_8_ RNA. (A) Representative isothermal titration calorimetry (ITC) thermogram and binding isotherm for the interaction between MPC04 and r­(CUG)_8_ RNA. The data are best-fitted by a multisite binding model, yielding dissociation constants of *K*_d1_ = 1 ± 0.3 nM and *K*_d2_ = 3 ± 2 nM, with apparent binding stoichiometries of *N*_1_ = 8 ± 2 and *N*_2_ = 9 ± 2. The upper panel shows the corrected heat rate as a function of time, and the lower panel shows the integrated heat change per injection with the corresponding fit. (B) Docking-based models illustrating plausible interaction modes of MPC04 with the r­(CUG)_8_ RNA duplex. Top panels show representative groove-associated binding poses in the minor and major grooves. Bottom panels show intercalation-compatible binding poses at CGUU and GCGC steps, illustrating stacking of the aromatic core between base pairs. These models are presented to illustrate feasible binding scenarios consistent with the experimental data and do not imply a unique binding mode.

In contrast, ITC experiments conducted under identical conditions using the DNA duplex revealed significantly weaker binding (Figure S11A). The fitted dissociation constants (*K*_d1_ = 450 ± 120 nM, *K*_d2_ = 310 ± 180 nM) indicate substantially reduced affinity compared with r­(CUG)_8_ RNA. This reduced binding affinity likely arises from structural differences between RNA and DNA duplexes, including variations in groove dimensions and backbone conformational flexibility, which can strongly influence ligand accessibility, binding efficiency, and binding mode. To further investigate structure–activity relationships, the binding properties of structurally related analogues MPC03 and MPC05 toward r­(CUG)_8_ RNA were also examined by ITC (Figure S11B–D). The binding isotherm for MPC03 was best described by a multisite binding model, yielding dissociation constants of *K*_d1_ = 180 ± 110 nM and *K*_d2_ = 180 ± 120 nM, corresponding to moderate affinity relative to MPC04. In contrast, MPC05 exhibited markedly weaker binding and was adequately fitted using a single-site binding model, with a dissociation constant of *K*_d_ = 2900 ± 300 nM.

The ITC-derived binding affinities correlate well with cellular assay results, in which MPC04 displayed the highest biological activity, followed by MPC03, whereas MPC05 showed neither appreciable cellular activity nor strong binding to r­(CUG)_8_ RNA. Collectively, these results establish MPC04 as the most potent and selective ligand among the compounds tested, exhibiting both high-affinity binding to CUG repeat RNA and corresponding cellular efficacy.

To visualize the potential interaction of MPC04 with a (CUG) repeat RNA, we employed a molecular modeling approach. The previously determined X-ray crystal structure of the (CUG) repeat RNA^36^ was first subjected to a short molecular dynamics simulation to relax the structure in water^37^ and then MPC04 was docked to the simulated RNA structure. Approximately 5% of the predictions show a potential minor groove binding conformation, with most sampled conformations located in the major groove. The chemical structure of MPC04 suggests that a positive terminal group, longer core structure, and higher flexibility provide an RNA binding interface. The MPC04 molecules can span a full turn of approximately 10 nucleotides on the RNA, thus allowing the piperazines to bind at regions with the highest negative charge density on the repeat RNA. In addition to groove binding, we visualized intercalation as a possible binding mode. Because rigid-body docking cannot accommodate the substantial backbone rearrangements required to form an intercalation site, we manually introduced intercalation sites into the relaxed RNA structure prior to docking to MPC04. The resulting docked poses suggest that intercalation is feasible: the two positively charged termini of MPC04 interact with the opposite strands of the duplex, while the central furan–benzene core stacks with the nucleobases at the intercalation site (Figure 7B). The docking scores of these intercalated poses fall within the same range as those for groove-bound poses, suggesting that intercalation can be an alternative binding mode (Figure S11E). To evaluate intercalation, fluorescence indicator displacement (FID) assays were performed using r­(CUG)_8_ RNA and MPC04. SYBR Safe was employed as an intercalation-sensitive dye, while Hoechst 33258 was used as a minor groove-binding indicator. Measurable displacement of both dyes by MPC04 (Figure S11F) suggests binding through either intercalative or groove-associated interactions. While these data do not allow unambiguous assignment of a single binding mode, they are consistent with the possibility that MPC04 can engage r­(CUG)_8_ RNA through more than one type of interaction. Taken together, this evidence suggests (i) that the piperazine rings are important for favorable interactions with the RNA backbone both for groove binding and/or intercalation; and (ii) the span of the MPC04 and the conformational flexibility arising from the multiring system promotes polar and nonpolar contacts on the grooves of the RNA. While qualitative and requiring additional detailed experiments, this analysis provides a tentative hypothesis regarding how the structural features of MPC04 might influence their interaction with the repeat RNA.

### Summary

Myotonic dystrophy type 1 (DM1) is a complex spliceopathy disease, with no approved therapeutics targeting the underlying toxic RNA disease mechanism. While previous work has demonstrated that small molecule diamidines have therapeutic potential for DM1,^20,22,26^ off-target effects and cellular toxicity have limited their application. In this work, we designed, synthesized, and tested a new series of small molecules, modified polycyclic compounds (MPCs), inspired by diamidine compounds. These MPCs are composed of three basic elements: a heterocyclic core, benzimidazole side groups, and functionalized end groups, allowing for various modifications. Splicing rescue analysis in a patient-derived DM1 fibroblast line identified two lead compounds (MPC03 and MPC04), which were validated in subsequent splicing rescue studies in two patient-derived DM1 myotube lines. While all three MPCs demonstrated the ability to rescue common DM1 mis-splicing events in patient-derived fibroblast and myotubes, in cell culture MPC04 had the best splicing rescue and showed reduced *DMPK* expression levels and increased *MBNL1* and *MBNL2* expression levels. These effects were observed in the low nanomolar range without significant effects on cell viability.

To better understand the mechanism of action and off-target effects of MPCs, lead candidates were examined by RNA-seq in DM1 myotubes and in a control myotube cell line. RNA-seq analysis confirmed that MPC04 was able to globally rescue dysregulated splicing events and gene expression patterns with limited off-target effects. Of the several thousand splicing events dysregulated in both DM1 myotubes treated with MPC04, over 50% were rescued by treatment at a low nM concentration (8 nM), with more events rescued in a dose-dependent manner up to 125 nM. Treatment in unaffected myotubes, which also did not affect viability, resulted in less than 100 events with a splicing change >30%, suggesting modest off-target effects. It is possible that MPC04 off-target activity and on-target rescue could occur through direct binding by MPC04 to affected transcripts. Direct binding to pre-mRNAs is how small molecules Risdiplam and Branaplam have been shown to rescue splicing dysregulation in hundreds of events effected in SMA with off-target activity as well.^38^ In the future, it will be interesting to compare the activities of MPCs to those of compounds like Risdiplam. While MPC03 and MPC04 compounds did demonstrate strong splicing rescue in patient-derived cell lines, a more modest effect was observed following short treatment of the HSA^LR^ mouse model. MPC03 and MPC04 showed modest splicing rescue in HSA^LR^ mice with subsequent reduction of *ACTA1* transgene expression levels and no change in endogenous *Dmpk* or *Mbnl* levels. Interestingly, MPC04 did not affect splicing and *Dmpk and Mbnl* expression levels in C2C12 mouse myoblasts but did affect *DMPK* and *MBNL* expression in unaffected human myotubes. These data suggest that our lead MPCs operate through binding or interacting with both short and long CUG repeat RNAs and their splicing rescue is closely tied to the reduction of CUG_exp_ RNA levels and possibly human-specific upregulation of MBNL proteins. While these data suggest a direct interaction with the *DMPK* RNA target, we cannot rule out the possibility that MPCs may also affect splicing by directly binding affected transcripts. Our molecular modeling of the MPC and CUG repeat RNA interaction suggests that this interaction disrupts MBNL sequestration, leading to increase in the expression of human *MBNL1* and *MBNL2* transcript levels and MBNL protein levels. The MPCs may bind CUG repeat RNA through intercalation, groove binding, or a combination of both binding modes.

The data presented herein show that the MPC compounds have interconnected effects on DM1 molecular pathology, including^1^ rescue of hundreds to thousands of dysregulated splicing events,^2^ reduction of *DMPK*/CUG_exp_ RNA levels, and^3^ modulation of MBNL protein and transcript abundance. While distinct, these effects could reflect interconnected actions. High affinity MPC binding to CUG_exp_ RNA disrupts sequestration of MBNL proteins, thereby restoring their availability for normal splicing regulation. The reduced CUG_exp_ RNA levels in turn further amplify this effect by decreasing the total amount of toxic RNA available to trap MBNL. Consistent with this model, MPC activity is absent in cells lacking CUG repeats, and splicing rescue strongly correlates with the degree of *DMPK* transcript reduction. Moreover, the observed increases in *MBNL1* and *MBNL2* transcript and protein levels likely reflect a secondary, compensatory response to restore MBNL function or reduce toxic RNA stress. Together, these findings suggest that MPCs initiate a cascade in which primary binding to CUG_exp_ RNA leads to both direct liberation of MBNL and indirect reinforcement of MBNL-dependent pathways.

In summary, we have identified a series of new multiringed molecules with nanomolar splicing rescue activity that show improved selectivity and viability over parental compounds.^20,22,26,39^ While there is therapeutic promise in this new MPC class, these compounds will require improvements in druglike properties (Table S1) and follow-up experiments to better understand their bioavailability and long-term effects across multiple DM1 mouse models. This further characterization will be assisted by the modular structure of the MPCs as we explore additional modifications to better understand the mechanism of action and improve solubility and deliverability of lead compounds. Given that DM1 is a member of a large and growing class of repeat expansion diseases that includes the leading cause of amyotrophic lateral sclerosis and corneal dystrophy, future work will also examine MPC potential across other repeat expansions.

## Materials and Methods

### General Methods for MPC Synthesis

The starting template (MPC01) was synthesized in the following manner: A solution of 2,5-bis­(4-formylphenyl)­furan (4, 105 mg, 0.377 mmol), *o*-phenylenediamine (5, 82 mg, 0.755 mmol), and 1,4-benzoquinone (82 mg, 0.755 mmol) in ethanol (70 mL) was heated at reflux for 4 h. The reaction mixture was cooled to RT, and the dark solid was collected by filtration, washed with cold ethanol and anhydrous ether, and dried to afford MPC01 (85 mg, 51% yield). A similar protocol was applied to a series of substrates (commercially available or prepared as previously described)^40^ to synthesize MPC02-10 in yields of 6–60%. All commercially available chemicals were used without further purification unless otherwise noted. Reactions were monitored by thin-layer chromatography using TLC silica gel 60-F_254_ plates. TLC plates were visualized by UV fluorescence (254 nM) or stained by cerium molybdate followed by heating. Purification of the reaction products was carried out by column chromatography using Siliaflash-P60 (40–63 μm) silica gel available from Silicycle.

### Patient-Derived Cell Culture

Primary fibroblast cell lines from skin biopsies of an affected 4 year old female DM1 patient (GM04601, DM1, Coriell Institute) and an unaffected 37 year old female individual (GM08400, Ctrl, Coriell Institute) were used for MPC screens. Fibroblasts were cultured in 1× MEM (Corning, cat. no. 10-010-CV) supplemented with 1x antibiotic–antimycotic (Gibco, cat. no. 15240062) and 15% heat-inactivated fetal bovine serum (HI-FBS, Corning, cat. no. 35-015-CV) at 37 °C with 5% CO_2_. Fibroblasts were seeded in 96-well plates at a density of 2 × 10^4^ cells/mL. After reaching ≥95% confluence, fibroblasts were treated with MPCs for 96 h in the same media and incubation conditions.

Primary myoblasts from two DM1 patients (DM1-A and DM1-B) and one unaffected individual (Ctrl) were used for testing MPC02, MPC03, and MPC04. DM1-A myoblast cells were obtained from muscle biopsies of a 45 year old female DM1 patient with approximately 2900 CTG repeats under a University of Florida-approved IRB protocol with informed consent from the subject.^41^ DM1-B myoblasts were obtained from a muscle biopsy of a 30 year old female DM1 patient with approximately 1317 CTG repeats. Ctrl cells originated from muscle biopsies of a 62 year old unaffected female and were sourced from Cell Applications (cat. no. 150-05a). All myoblasts were cultured with Skeletal Muscle Cell Growth Medium-2 BulletKit (SkGM-2, Lonza, cat. no. CC-3246) media. Myoblast growth media was supplemented with SkGM-2 SingleQuots (Lonza, cat. no. CC-3244), which provided appropriate components necessary for proliferation of skeletal muscle myoblasts. Myoblasts were seeded at a density of 1 × 10^5^ cells/mL in 12-well plates. When cells reached ≥90% confluence, they were differentiated in DMEM/F-12 50/50 (Corning, cat. no. 10-90-CV) media supplemented with 2% horse serum albumin (HSA, Corning, cat. no. 35-030-CV) for 96 h. After differentiation, cells were treated with MPCs in SkGM-2 media with previously mentioned added supplements for 96 h.

### MPC Preparation for Tissue Culture Experiments

Fibroblast and myotube cell models were treated with MPCs for 96 h unless specified otherwise. MPCs were dissolved into 99.9% dimethyl sulfoxide (DMSO, Sigma-Aldrich, cat. no. D2650) to create 10 mM stock concentrations. Stocks were at −80 °C, and no reduction in MPC activity was observed after repeated freeze–thaw cycles. Prior to use, MPCs were thawed in 37 °C water baths for 5 min, sonicated for 5 min, vortexed for 30 s, and briefly centrifuged for 3–5 s on a benchtop centrifuge. 10 mM stock concentrations of MPCs were diluted to μM concentrations in DMSO. Then, μM concentrations of MPC and DMSO were diluted by 1000-fold in treatment media. All experiments included DMSO-treated cells as vehicle controls, and the final concentration of DMSO at 0.1% (v/v) was consistent throughout treatment concentrations.

### Cell Viability Assay

Cell viability was quantified using a resazurin-based assay (PrestoBlue Cell Viability Reagent, ThermoFisher, cat. no. 13261) following the manufacturer’s protocol. This viability assay works by measuring how living cells reduce the resazurin reagent into a fluorescent, highly detectable product, providing a readout proportional to the overall cellular metabolic activity. Cells were treated for 20 min at 37°C, allowing time for the reagent to become reduced by metabolically active cells. Absorbance was read at 570 nM, and each replicate was made relative to the average absorbance of the 0.1% DMSO (v/v) vehicle control-treated patient-derived DM1 myotubes. Significant changes between conditions were determined by a one-way ANOVA and Dunnett’s multiple comparison test, with the DM1-treated vehicle control group used as a baseline.

### Animal Husbandry and Treatment Approach

All animal husbandry was carried out at the Center for Neurogenetics at the University of Florida under an approved IACUC protocol by E. T. Wang. HSA^LR^ mice and FVB/NJ (wild type, WT) mice were used for treatment by MPC03 and MPC04. MPCs were dissolved in a 1:1 (v/v) mixture of DMSO and PBS and delivered by IP injection at 20 mg/kg to HSA^LR^ and WT mice. Control mice for WT and HSA^LR^ groups were treated with vehicle control (1:1 DMSO-PBS) at the same time with equal volumes of DMSO-PBS as the 25 mg/kg treated mice. In total, four *n* = 4 mice were subjected to each condition (vehicle, MPC03 25 mg/kg, MPC04 25 mg/kg) for both WT and HSA^LR^ mouse models. Consideration for sex differences was accounted for by balancing the WT and HSA^LR^ mice with 2 males and 2 females for each condition, with exception to the HSA^LR^ vehicle control treatment group, which was composed of 3 males and 1 female.

### RNA Extraction

RNA was extracted from fibroblasts using the NucleoSpin 96 RNA extraction kit (TakaraBio, cat. no. 740709.4) following the manufacturer’s protocol including an on-column DNase1 treatment for genomic DNA degradation. RNA was extracted from myotube cells using the Quick-RNA Miniprep Kit (Zymo Research, cat: R1055) with on-column DNase I treatment following the manufacturer’s protocol. Mouse tibialis anterior muscle tissue was pulverized with 1.5 mm zirconium beads (Genesee Scientific, cat. no. 31-212Z15) and TRIzol reagent (Thermo Fisher, cat. no. 15596026) in 2 mL tubes with a high-speed bead beater (Omni International, Bead Ruptor 12, cat. no. 19-050A). The Direct-zol RNA Miniprep Plus (Zymo Research, cat. no. R2070) was then followed, including an on-column DNase I treatment, to purify RNA. RNA yields were measured using a spectrophotometer measuring the 260/280 absorbance ratio of RNA to DNA (NanoDrop One^C^, Thermo Scientific, cat. no. ND-ONE-W). All RNA was stored at −80 °C for downstream use.

### Reverse Transcription and PCR Alternative Splicing Isoform Analysis

RNA samples were reverse-transcribed using the SuperScript IV platform (Thermo Scientific, cat. no. 18090010). The following modifications to the manufacturer’s protocol were made: total RNA input varied from 100 to 500 ng, 50 μM random hexamers were sourced from IDT (cat. no. 51-01-18-26), and a 30 min 50 °C incubation time was used. The resulting cDNA was used for polymerase chain reaction (PCR) using NEB *Taq* 2x Master Mix (NEB, cat. no. M0270L). PCR primer sequences and annealing temperatures (*T*_m_) are reported in Table 2. The manufacturer’s conditions were followed for 25 μL reactions with 32 total cycles. PCR product was measured via capillary electrophoresis (Fragment Analyzer, Agilent Technologies, cat. no. M5311AA) using the DNF-905 kit (cat. no. DNF-905-0500) to measure product ranging from 1 to 500 bp. To quantify PSI, the concentration of inclusion product and exclusion product, represented by relative fluorescence unit (RFU) values, were used after data processing using Agilent Technologies ProSize 2.0 File Handler software. PSI was calculated by dividing numerator, the RFU value of the inclusion product, by the denominator, the sum of RFU values for the inclusion and exclusion products and multiplying the quotient by 100 (eq 1).

**Table 2:** Splicing Primers^a^

splicing primers (5′–3′)	
*FLNB* exon 31 forward	GCTTCGGTGGTGTTGATATTC
*FLNB* exon 31 reverse	GTCACTCACTGGGACATAGG
*INSR* exon 11 forward	CCTGTCCAAAGACAGACTCTCAGATCCTG
*INSR* exon 11 reverse	GTCGAGGAAGTGTTGGGGAAAGC
*MBNL1* exon 5 forward	AGGGAGATGCTCTCGGGAAAAGTG
*MBNL1* exon 5 reverse	GTTGGCTAGAGCCTGTTGGTATTGG
*NUMA1* exon 16 forward	AAGTATGAGGGTGCCAAGGT
*NUMA1* exon 16 reverse	CTTCAGCTTCTGCTGCTGCA
*Atp2a1* exon 22 forward	GCTCATGGTCCTCAAGATCTCAC
*Atp2a1* exon 22 reverse	GGGTCAGTGCCTCAGCTTTG
*Clcn1* exon 7a forward	TGAAGGAATACCTCACACTCAAGG
*Clcn1* exon 7a reverse	CACGGAACACAAAGGCACTG
*Mbnl1* exon 5 forward	GCTGCCCAATACCAGGTCAAC
*Mbnl1* exon 5 reverse	TGGTGGGAGAAATGCTGTATGC

a Splicing primers are listed by row. Primers of the same name were paired in PCR experiments by their “Forward” and “Reverse” designations. Sequences of primers are listed in a 5′–3′ orientation (right to left). Human splicing primers have gene names in all capital letters. Mouse splicing primers have gene names with the first letter capitalized.

### Real-Time Quantitative PCR Analysis

Real-time quantitative PCR (RT-qPCR) was performed in two steps. First, RNA was reverse-transcribed as described. RNA input concentrations were identical across replicates and experiments. Then, cDNA was amplified using the PowerUp SYBR 2x Master Mix assay (Applied Biosystems, cat. no. A25777) and appropriate qPCR primers (Table 3) following the manufacturer’s protocol for 10 μL reactions in a 384-well plate. Reactions were operated by a CFX384 Touch Real-Time PCR system (Bio-Rad, cat. no. 1855484). Results were analyzed following the delta–delta Ct method.^42^ The expressions of *DMPK*, *MBNL1*, and *MBNL2* were normalized to *GAPDH* and made relative to the average of vehicle control-treated samples. The same approach was used to quantify mouse gene expression levels using the appropriate qPCR primers (Table 3). *Dmpk*, *Mbnl1*, *Mbnl2*, and *ACTA1* were normalized to *Gtf2b*.

**Table 3:** Gene Expression Primers^a^

qPCR primers (5′–3′)	
*DMPK* forward	CACGTTTTGGATGCACTGAGAC
*DMPK* reverse	GATGGAGGGCCTTTTATTCGCG
*MBNL1* forward	CGCAGTTGGAGATAAATGGACG
*MBNL1* reverse	CACCAGGCATCATGGCATTG
*MBNL2* forward	CCTGGTGCTCTTCATCCTTTAC
*MBNL2* reverse	GTGAGAGCCTGCTGGTAGTG
*GAPDH* forward	AATCCCATCACCATCTTCCA
*GAPDH* reverse	TGGACTCCACGACGTACTCA
*ACTA1* forward	GGAGCGCAAATACTCGGTG
*ACTA1* reverse	CATTTGCGGTGGACGATGG
*Mbnl1* forward	ACAGAAGTTAATGCGGACAGA
*Mbnl1* reverse	GATGAGCAAACCGACAGTCA
*Dmpk* forward	CTGCTCGACCTTCTCCTGG
*Dmpk* reverse	CACGCCCGATCACCTTCAA
*Gtf2b* forward	CTTCATGTCCAGGTTCTGCTCC
*Gtf2b* reverse	GGAACCAAGTCCAGCTCCAC

a qPCR primers are listed by row. Primers of the same name were paired in qPCR experiments by their “Forward” and “Reverse” designations. Sequences of primers are listed in a 5′–3′ orientation (right to left). Human qPCR primers have gene names in all capital letters. Mouse qPCR primers have gene names with the first letter capitalized.

### Fluorescence In Situ Hybridization

DM1-B and control myoblasts were cultured as described above in 8-chamber slides (Corning BioCoat), differentiated into myotubes for 96 h, and then treated with MPC04 or DMSO for 96 h. Myotubes were then fixed with 4% PFA (made in DEPC-treated water and 1× PBS) for 15 min, rinsed with PBS, and permeabilized using prechilled 70% ethanol. Myotubes were rehydrated in 2× SSC buffer and prehybridized in the presence of tRNA (Thermo Fisher cat. no. AM7119) at 37 °C for 30 min. Slides were hybridized with a Cy3-(CAG)_8_ probe (IDT) overnight at 50 °C, washed with prewarmed 2× SSC at 42 °C, and mounted using ProLong Diamond Antifade mountant with DAPI (Thermo Fischer cat. no. P36962). Myotubes were imaged by using a confocal microscope (Zeiss LSM 980) with a 40× objective. Nuclei and foci were counted by using CellProfiler and a modified version of its speckle counting pipeline. At least 90 nuclei were counted per replicate.

### Western Analysis

Patient-derived myotubes were treated as described above with MPC04, drug media was removed, and cells were briefly washed with 1xPBS. Cells were lysed with 100 μL RIPA buffer (Thermo Scientific, cat. no. 89901) supplemented with cOmplete Protease Inhibitors (Roche, cat. no. 11697498001) for 15 min at 4 °C. Soluble protein fractions were separated from insoluble fraction through centrifugation at 14,000 rpm for 15 min at 4 °C. The supernatant was separated and stored at −20 °C for Western blot analysis. The Pierce BCA Protein Assay kit (Thermo Scientific, cat. no. 23225) was used to determine protein concentration for each sample. Standards were measured in triplicate, and samples were measured in duplicate in a 96-well, clear bottom plate. Background was removed from each measurement in Excel, and a linear standard curve was generated to calculate the total concentration of soluble protein present within each sample.

A minimum of 13 μg of protein was used per sample and combined with 6x Laemelli buffer (Thermo Scientific, cat. no. J61337-AC) and molecular grade water. Protein samples were denatured at 95 °C for 5 min, centrifuged with a desktop centrifuge, and held at 4 °C or on ice until loading onto 4–12% Criterion XT Bis-Tril Protein gels (Bio-Rad, cat. no. 3450125). Proteins were separated by size in 1xMOPS (Bio-Rad, cat. no. 1610788) at 80 V for 20 min followed by 125 V for 90 min. Separated proteins were transferred onto PVDF membrane (EMD Millipore, cat. no. IPFL00005) in 1xNuPAGE transfer buffer (Thermo Scientific, cat. no. NP00061) for 2 h at 0.3 A and 4 °C. Membranes were blocked using Intercept (TBS) Blocking Buffer (LI-COR, cat. no. 927-60001) for 1 h at RT. Membranes were then washed, 3 times with 1x TBS-T at 5 min incubation periods, and then probed with rabbit-*anti*-MBNL1 (EMD Millipore, cat. no. ABE241; 1:400 dilution) and mouse-*anti*-GAPDH (Abcam, cat. no. ab8245; 1:5000 dilution) in Intercept Blocking Buffer (LICORbio, cat. no. 927-70001) overnight at 4 °C. Then, membranes were incubated with secondary antibodies at RT for 1 h. MBNL1 was probed with donkey-antirabbit IRDye@680 (LICORbio, cat. no. 926-68073; 1:7500 dilution) and goat-antimouse IRDye@800 (LICORbio, cat. no. 926-32210; 1:7500 dilution). After secondary incubations, membranes were washed 3 times with 1× TBS-T at 5 min incubation periods. Fluorescently labeled proteins were measured using the ChemiDoc MP Imaging System (Bio-Rad, cat. no. 12003154), and the image file was exported. Image processing was performed with FIJI’s ImageJ software (v1.53s). Background intensity was subtracted from GAPDH and MBNL1 band intensity; each MBNL1 signal was normalized to its respective GAPDH signal, and all samples were normalized to the average of the vehicle control-treated samples.

### Library Generation, RNA-seq, and Alternative Splicing and Gene Expression Analysis

For each sample, from Control, DM1-A, and DM1-B myotubes treated with vehicle control (0.1% DMSO v/v) or MPC04 at various concentrations, 100 ng of total RNA was used to generate RNA-sequencing libraries. RNA integrity numbers (RINs) were measured via capillary gel electrophoresis with the DNF-471 (Agilent, cat. no. 5191-6572) kit, which provides an RIN value. All samples had an RIN value greater than 7. RNA samples were prepared for sequencing using the NEBNext Ultra II Direction RNA library Prep Kit for Illumina (NEB, cat. no. E7760L) with rRNA depletion via the NEBNext rRNA Depletion Kit (NEB, cat. no. E6310X). The following changes were made to the manufacturer’s protocol: 40x adaptor dilutions were made, bead incubations were all performed at RT, and 15 cycles of library amplification were used. Libraries were quantified for quality and yield using the NEBNext Library Quant Kit (NEB, cat. no. E7630) and capillary gel electrophoresis using the DNF-474 kit (Agilent, cat. no. DNF-474-0500). Libraries were pooled in equimolar concentrations and sequenced on the Illumina NextSeq 2000 (Illumina, cat. no. 20038897) with the NextSeq 1000/2000 P2 XLEAP-SBS Reagent Kit (200 cycles, 2 × 150bp) (Illumina, cat. no. 20100986). A total sequencing depth of ∼62 million paired end reads per sample was achieved.

Subsequent FASTQ files were assessed for quality and total number of reads using FASTQC (v0.11.9). Paired-end reads were trimmed, and low-quality reads were filtered using FASTP (v0.23.4) with default settings.^43^ Sequence alignment to the human reference genome (GRCh38.p13) was performed by HISAT2 (v2.2.1).^44^ SAM file output was reformatted to BAM format using SAMtools (v1.17).^45^ Alternative splicing analysis was performed with rMATS-turbo (v4.2.0),^46^ with BAM files as input and the optional flags “—allow-clipping” and “—variable-read-length”. All comparisons of diseased or treated groups were made against the control groups. The resulting JCEC files were used for the analysis of SE events only. To match SE events across data sets, a unique ID was generated for each event consisting of the upstream, downstream, and regulated exon coordinates, Ensembl ID, and gene symbol. SE events matched across data sets were processed by removing rows that contained missing values across samples from each baseline, untreated group (Ctrl, DM1-A, and DM1-B), and by retaining events that had standard deviation among groups ≤20%. SE events were considered dysregulated when the absolute value of the inclusion level difference of control, untreated, and DM1 affected, untreated myotubes was ≥0.1 with an FDR value ≤0.05. Exon inclusion level values measured by rMATS-turbo for each replicate within each group were transformed to PSI by multiplying reported values by 100. We assessed whether treatment with MPC04 changed dysregulated splicing levels by calculating percent rescue using PSI from each respective group as input values.

SE events that showed a ≥10% rescue were labeled “Rescue”. SE events with a ≤ −10% rescue, where splicing shifted further away from control levels after treatment, were labeled “opposite-direction”. SE events, which did not meet our cutoff threshold to be considered dysregulated between control and DM1 affected myotubes but did significantly change after MPC04 treatment (FDR ≤0.05, absolute exon inclusion level difference ≥0.1), were labeled as “Off-target”.

Differential expression analysis was performed using the HISAT2, StringTie, and prepDE.py3 ^44^ pipeline to generate gene count matrices. R (v4.3.1) was used to run DESeq2 (v1.40.1), which took gene count matrices as input. DESeq2-normalized counts were used to measure gene expression for each comparison. Genes were differentially expressed between groups if the absolute log2 fold change value was ≥2 and the adjusted *p*-value was ≤0.05. Gene ontology (GO) analysis was performed using the Enrichr webtool.^30–32^

### Isothermal Titration Calorimetry

Isothermal titration calorimetry (ITC) was performed by using a Nano ITC instrument (TA Instruments) to investigate the binding interaction between MPC04 and RNA or DNA duplexes. The RNA sequence used was 5′-CUG­CUG­CUG­CUG­CUG­CUG­CUG­CUG-3′, and the DNA sequence was 5′-CTG­CTG­CTG­CTG­CAG­CAG­CAG­CAG-3′. Stock solutions of RNA and DNA were prepared in 1× sodium cacodylate buffer (10 mM sodium cacodylate, 25 mM NaCl, and 1 mM EDTA, pH 7.0). The oligonucleotides were annealed by heating at 95 °C and slowly cooling to RT overnight. A volume of 190–200 μL of 25 μM RNA or DNA duplex was loaded into the sample cell. MPC04 was initially dissolved at 50 mM in 1× sodium cacodylate buffer without EDTA, as EDTA caused molecule precipitation at high concentrations. A 4 mM working solution was freshly prepared before each experiment. To avoid buffer mismatch, an equivalent amount of 1× sodium cacodylate buffer containing EDTA was added to the working solution before it was loaded into the syringe. Each titration consisted of 12 injections of 4 μL of 1 mM MPC04/MPC03/MPC05 with a 250 s interval between injections. Data were analyzed using NanoAnalyze version 4.1.0.1 (TA Instruments). The heat released or absorbed during each injection was calculated by integrating the area under the power-versus-time curve. Blank titrations, in which MPC04/MPC03/MPC05 was injected into the buffer alone under identical conditions, were subtracted from the experimental data to obtain corrected heat values. All experiments were performed in at least triplicate, and errors are reported as the standard deviation of the measurements. Binding isotherms were fitted by either multisite or one-site binding model.

### Fluorescence Intercalator Displacement Assay

r­(CUG)_8_ RNA was dissolved in 1× sodium cacodylate buffer (10 mM sodium cacodylate, 25 mM NaCl, and 1 mM EDTA, pH 7.0) and annealed by heating at 90 °C for 10 min, followed by slow cooling to RT overnight. Fluorescence indicator displacement assays were performed using either a SYBR Safe (Thermo Fisher Scientific) or a Hoechst 33258 as fluorescent probes. SYBR Safe was used as an intercalation-sensitive dye, while Hoechst 33258 served as a minor groove-binding indicator. Fluorescence measurements were recorded using an excitation wavelength of 500 nm and an emission wavelength of 530 nm for SYBR Safe, and an excitation wavelength of 352 nm with an emission wavelength of 454 nm was recorded for Hoechst 33258. Each assay solution contained 2 μM r­(CUG)_8_ RNA, 100 nM fluorescent dye, and increasing concentrations of MPC04 (0–10 μM). Fluorescence intensity (*F*) was measured after equilibration at RT using a plate reader, and the percentage of fluorescent intercalator displacement (%FID) was calculated using eq 4:

4 $$\%FID=100\%-\left(100\%\times \frac{F}{F_{0}}\right)s$$

### Simulation and Modeling of MPC Binding

Molecular docking was performed to gain insight into the interaction of the MPCs with CUG repeat RNA. A previously solved X-ray crystal structure of the CUG RNA (PDB ID: 5MWI)^47^ was used as the receptor. First, molecular dynamics simulation (MDS) was performed using GROMACS 2019.4 ^37^ to relax the structure in water. The AMBER99 force field with Chen-Garcia^48^ modifications was used to simulate the RNA. The simulation was carried out in TIP4P-Ewald^49^ water with 1 M KCL. The MDS incorporated a leapfrog algorithm with a 2 fs time step to integrate the equations of motion. The system was maintained at 320 K with the velocity rescaling thermostat^50^ and at 1 atm pressure using the Berendsen barostat.^51^ Long-range electrostatic interactions were calculated using the particle mesh Ewald (PME) algorithm with a real space cutoff of 1.0 nm.^52^ Lennard-Jones interactions were truncated at 1.0 nm. The LINCS algorithm was used to constrain the motion of hydrogen atoms bonded to heavy atoms.^53^ The system was subjected to a short energy minimization to prevent any overlap of atoms, followed by a 13 ns molecular dynamics simulation. Coordinates were stored every 2 ps for further analysis. The simulations were visualized using PyMOL^54^ and visual molecular dynamics (VMD)^55^ and analyzed using tools from GROMACS.^56^ The simulation trajectory frames were clustered using the RMSD (root-mean-square deviation) to identify the most dominant conformation of the RNA, which was subsequently utilized for molecular docking. To explore intercalation as a potential binding mode, RNA structure-containing predefined intercalation sites were generated using the w3DNA web server.^57^ Owing to the repetitive nature of the CUG repeat RNA, two unique intercalation sites are possible, bordered by either UU.GC or a GC.GC base pair steps. These sites were introduced near the center of the CUG8 RNA structure for subsequent docking. The docking study used the molecular operating environment (MOE) software.^58^ The small molecule MPC04 was prepared and assigned protonation states at a pH of 7.4. Molecular docking was performed using the triangle matching and induced fit algorithm for placement with 50 initial and 20 refined poses for each ligand. The docking scores provided by MOE (representative of the binding affinity of the small molecules to the RNA) were compared, and the predicted structures of the RNA/MPC complex were visualized in PyMOL to gain insights into the interaction of MPC04 with the CUG repeat RNA.

### Statistics

All statistical comparisons for RT-PCR and RT-qPCR were performed in GraphPad Prism (GraphPad Prism, v10.3.1). For sets of data with only two groups, Mann–Whitney U tests were performed, with a two-sided *p*-value <0.05 considered significant. For sets of data with greater than two groups, an ordinary one-way ANOVA test was performed to determine whether there were significant differences among groups. Post hoc pairwise comparisons were conducted using Tukey’s multiple comparison test to identify specific group differences. Statistical significance was set at *p* < 0.05. To assess the relationship between MPC04 dose and the effect on the expression of *DMPK* and *MBNL1* exon 5 inclusion or *NUMA1* exon 16 inclusion, a Pearson correlation analysis was conducted. The Pearson correlation coefficient (*r*) was calculated to determine the strength and direction of the linear association between *DMPK* expression and exon inclusion for *MBNL1* exon 5 or *NUMA1* exon 16. A *p*-value of <0.05 was considered statistically significant. While performing the RNA-seq analysis, the significance values computed by DESeq2 or by rMATS-turbo were used as reported by the software.

## Supplementary Material



## Data Availability

All RNA-seq data are uploaded to the National Center for Biotechnology Information’s Sequence Read Archive (BioProject: PRJNA1178131; SRA Study: SRP541008).

## Notes

Measures of druglike properties (Table S1); MPCs do not reduce fibroblast viability at the concentrations in which they rescue mis-splicing (Figures S1); modifications to MPC orientation and core size lead to loss of mis-splicing rescue (Figure S2); characterization of the DM1-B myotube cell line (Figure S3); MPC04 treatment reduces CUG ribonuclear foci in DM1-B myotubes (Figure S4); MPC04 treatment increases *MBNL1* and *MBNL2* transcripts in DM1 myotubes (Figure S5); MPC04 treatment affects MBNL1 protein expression in DM1 myotubes (Figure S6); splicing change following MPC treatment of unaffected control myotubes (Figure S7); gene expression effects from MPC04 treatment (Figure S8); MPC04 does not affect splicing or gene expression in C2C12 mouse myoblasts (Figure S9); MPC04 reduces *DMPK* transcript levels and increases both *MBNL1* transcript and MBNL1 protein levels in unaffected control myotubes (Figure S10); and ITC analysis of MPC analogues and experimental support for multiple binding modes (Figure S11) (PDF)

## Nomenclature

DM: myotonic dystrophy
DM1: myotonic dystrophy type 1
PSI: percent spliced in
S^2*^: splicing score
DMSO: dimethyl-sulfoxide
PBS: phosphate buffer saline
DM1-A: myotonic dystrophy type 1 myotube cell line A long repeat
DM1-B: myotonic dystrophy type 1 myotube cell line B moderate repeat
WT: wild type
*HSA* ^LR^: human skeletal actin long repeat
RT: reverse transcription
PCR: polymerase chain reaction
RT-qPCR: real-time quantitative PCR
HSA: horse serum albumin
HI-FBS: heat inactivated fetal bovine serum
MPC: modified polycyclic compound
MPC01: 2,5-bis­(4-(1*H*-benzo­[*d*]­imidazole-2-yl)­phenyl)­furan
MPC02: 2,5-bis­(5-(1*H*-benzo­[*d*]­imidazole-2-yl)­pyridine-2-yl)­furan
MPC03: 2,5-bis­(4-(6-(4-methylpiperazin-1-yl)-1*H*-benzo­[*d*]­imidazole-2-yl)­phenyl)­furan
MPC04: 2,5-bis­(5-(6-(4-methylpiperazin-1-yl)-1*H*-benzo­[*d*]­imidazole-2-yl)­pyridine-2-yl)­furan
MPC05: 2,5-bis­(3-(6-(4-methylpiperazin-1-yl)-1*H*-benzo­[*d*]­imidazole-2-yl)­phenyl)­furan
MPC06: 6-(4-methylpiperazin-1-yl)-2-(3-(5-(4-(6-(4-methylpiperazin-1-yl)-1*H*-benzo­[*d*]­imidazole-2-yl)­phenyl)­furan-2-yl)­phenyl)-1*H*-benzo­[*d*]­imidazole
MPC07: 3,7-bis­(4-(6-(4-(methylpiperazin-1-yl)-1*H*-benzo­[*d*]­imidazole-2-yl)­phenyl)­dibenzo­[*b*,*d*]­furan
MPC08: 2,5-bis­(6-(4-methylpiperazin-1-yl)-1*H*-benzo­[*d*]­imidazole-2-yl)­furan
MPC09: 2,5-bis­(4-(4-methylpiperazin-1-yl)­phenyl)­furan
MPC10: 2,5-bis­(3-fluoro-4-(4-methylpiperazin-1-yl)­phenyl)­furan
