# Supplementary material for: Modified Polycyclic Compounds Rescue Mis-splicing in Myotonic Dystrophy Type 1 Disease Models
Source: ACS Chem Biol. 2026 Feb 20;21(3):502–18. doi: 10.1021/acschembio.5c00790 (PMC13010256; doi:10.1021/acschembio.5c00790)
Supplement: Supplementary file 1 [file cb5c00790_si_001.pdf]

Supporting Information

Modified polycyclic compounds rescue mis-splicing in myotonic dystrophy type 1 disease models

[¥]Jesus A. Frias[1,2], [¥]Sawyer M. Hicks[1,2], Hormoz Mazdiyasni[1], Subodh K. Mishra[1], Kahini Sarkar[1], Clara Yeboah[1,2], Noah M. LeFever[1], Marina M. Scotti[3], Hana Zeghal[1], Naomi Brandt[1], Sweta Vangaveti[1], Pramita Chakma[1,4], Ting Wang[1], Tammy S. Reid[1], Omari McMichael[5,6], Christopher Crumbaugh[5,6], Marina Provenzano[5,6], Melissa A. Hale[5,6], John D. Cleary[1], Nicholas E. Johnson[5,6], Eric T. Wang[3], Kaalak Reddy[1,2], and J. Andrew Berglund[1,2*]

[1]The RNA Institute, College of Arts and Sciences, University at Albany, State University of New York, Albany, NY 12222, USA

[2]Department of Biological Sciences, College of Arts and Sciences, University at Albany, State University of New York, Albany, NY 12222, USA

[3]Department of Molecular Genetics & Microbiology & the Center for Neurogenetics, College of Medicine, University of Florida, Gainesville, FL 32610, USA

[4]Department of Chemistry, College of Arts and Sciences, University at Albany, State University of New York, Albany, NY 12222, USA

[5]Department of Neurology, Virginia Commonwealth University, Richmond, VA 23284, USA

[6]Center for Inherited Myology Research, Virginia Commonwealth University, Richmond VA 23298, USA

[¥]Co-first authors; * Corresponding author: aberglund@albany.edu

Table S1. Measures of druglike properties of MPC01-MPC10.

	Molecular Weight (g/mol)	Number of hydrogen bond Acceptors	Donors	MLOGP	WLOGP	TPSA (Å²)
MPC01	452.51	3	2	4.51	7.70	70.50
MPC02	454.48	5	2	2.50	6.49	96.28
MPC03	721.72	3	4	-3.63	-3.15	85.86
MPC04	723.70	5	4	-5.50	-4.36	111.64
MPC05	721.72	3	4	-3.63	-3.15	86.86
MPC06	721.72	3	4	-3.63	-3.15	85.86
MPC07	821.84	3	4	-2.12	-0.84	85.86
MPC08	569.53	3	4	-5.37	-6.48	85.86
MPC09	489.48	1	2	-4.44	-6.23	28.50
MPC10	525.46	3	2	-3.71	-5.11	28.50

MLOGP: Moriguchi octanol-water partition coefficient; WLOGP: Wildman-Crippen octanol-water partition coefficient; TPSA: Topological polar surface area.

To determine the druglike properties of MPC01-MPC10, the webtool SwissADME was used to calculate the following measures with filters applied: (1) Lipinski's rule of five (MW ≤ 500, number of H-bond acceptors ≤10, number of H-bond donors ≤5, calculated log P ≤5) in which MPC03-08 &10 were not optimal (shaded); (2) Verber (TPSA ≤140) and Egan (TPSA ≤131.6) filters in which all MPCs passed; (3) Egan filter (WLOGP ≤5.88) in which MPC01 & MPC02 failed (shaded); and (4) Ghose filter (− 0.4 ≤ WLOGP ≤ 5.6) in which all MPCs failed (shaded).

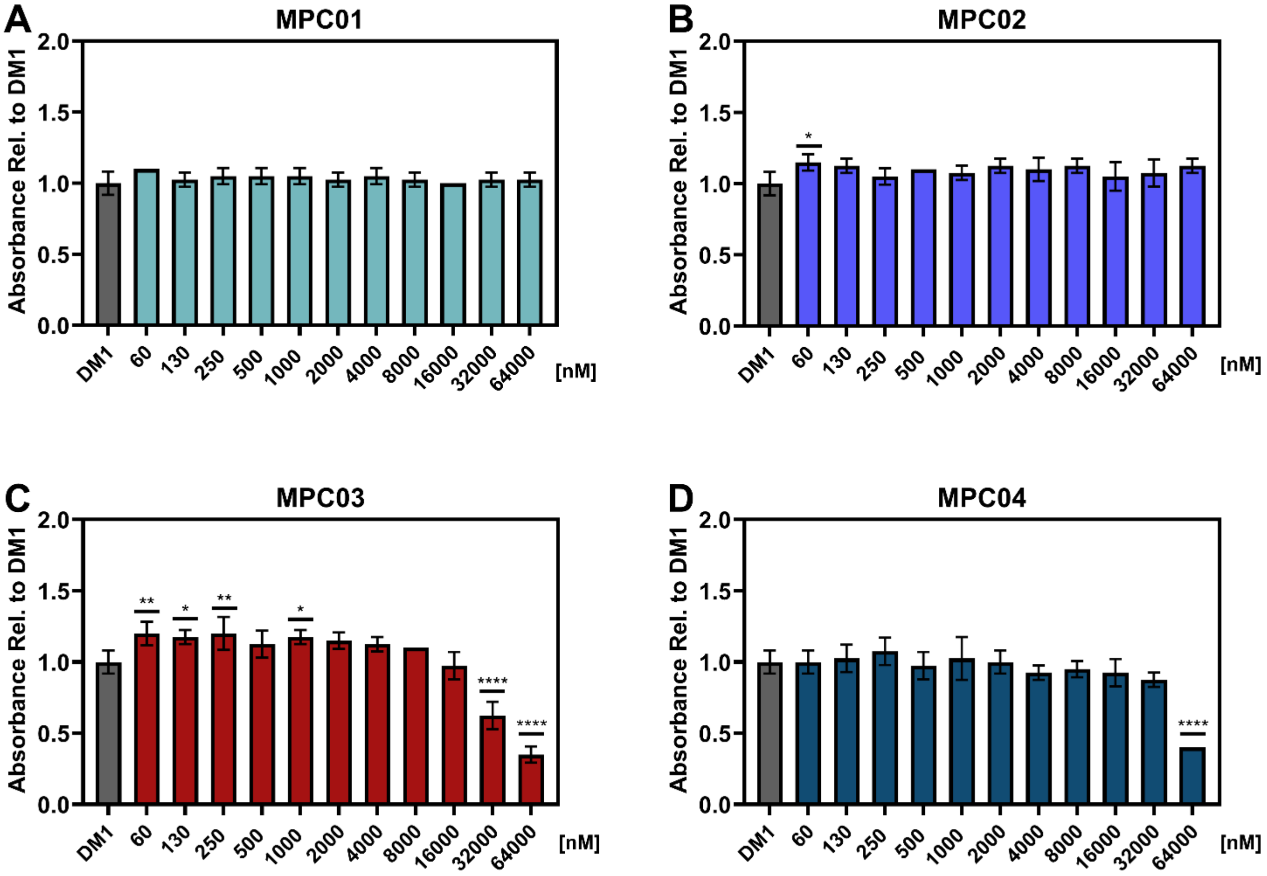

Figure S1. MPCs do not reduce fibroblast viability at the concentrations in which they rescue mis-splicing. HS Presto blue assay in DMSO and MPC treated DM1 fibroblasts shows that MPC01 (**A**), MPC02 (**B**), MPC03 (**C**), and MPC04 (**D**) do not have detrimental effects on cell viability at the concentrations in which mis-splicing rescue is observed. Adjusted-P values were determined with one-way Anova and Dunnett's multiple comparisons to the untreated DM1 values (n=4 for all treatments, mean± SEM. (P-adj < 0.05 = *, < 0.01**, < 0.0005***, < 0.0001****). Absorbance at 570 nm normalized to 600 nm and made relative to untreated.

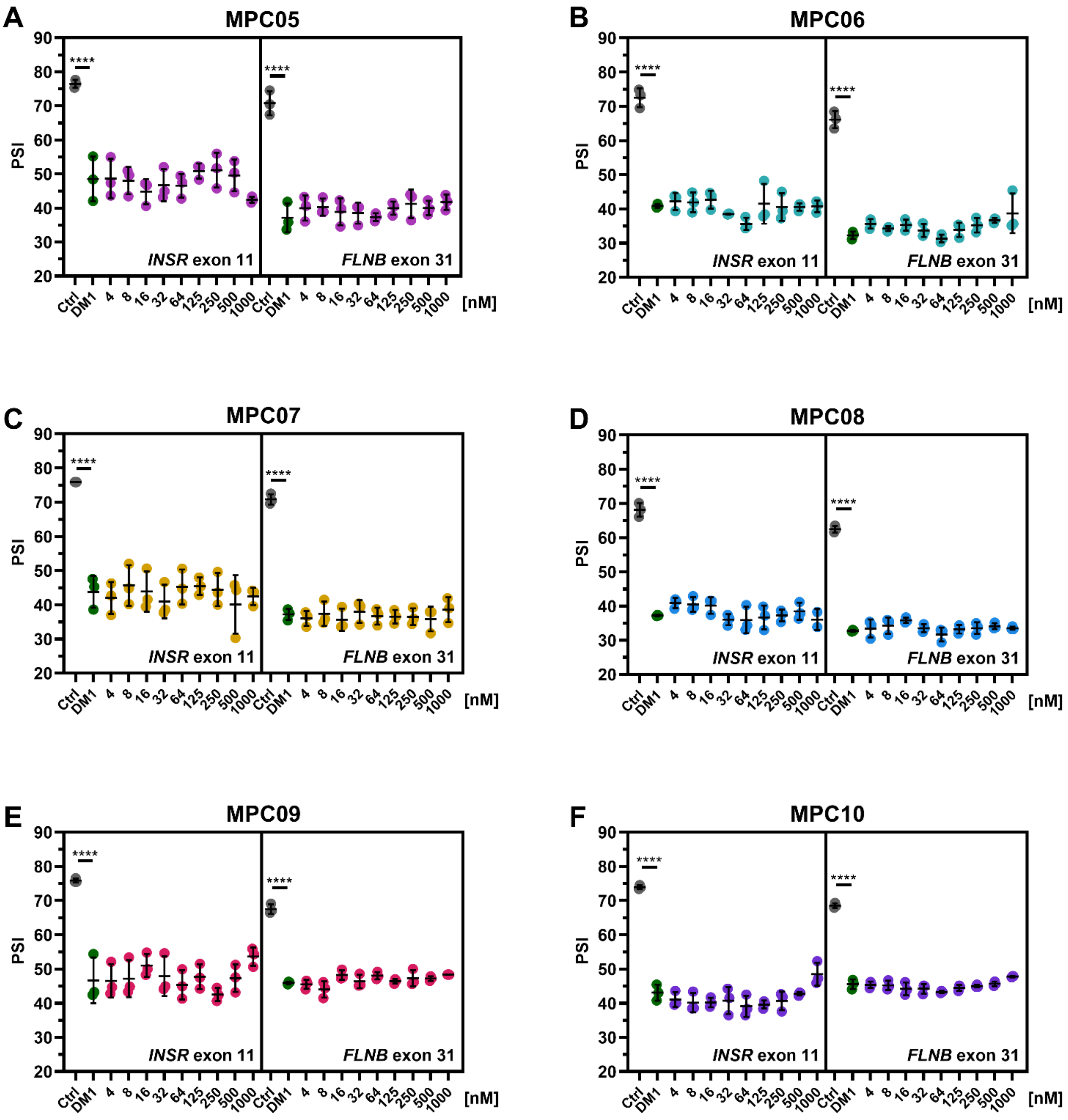

Figure S2. Modifications to MPC orientation and core size lead to loss of mis-splicing rescue. RT-PCR isoform analysis of cassette exons to assess effects on mis-splicing rescue from modifications to MPC core. The ΔPSI between Control and DM1 fibroblasts DMSO treated and treated with (**A**) MPC05, (**B**) MPC06, (**C**) MPC07, (**D**) MPC08, (**E**) MPC09, (**F**) or MPC10 was used to assess mis-splicing rescue. Mean ± standard error of the mean, Ctrl= Unaffected individual fibroblasts (n=24), 0= DMSO treated DM1 fibroblasts (n=32), MPC treated DM1 cells had n=3 for each concentration. One-way Anova with Dunnet's multiple comparisons to DMSO treated fibroblasts (P-adj < 0.05 = *, < 0.01**, < 0.0005***, < 0.0001****).

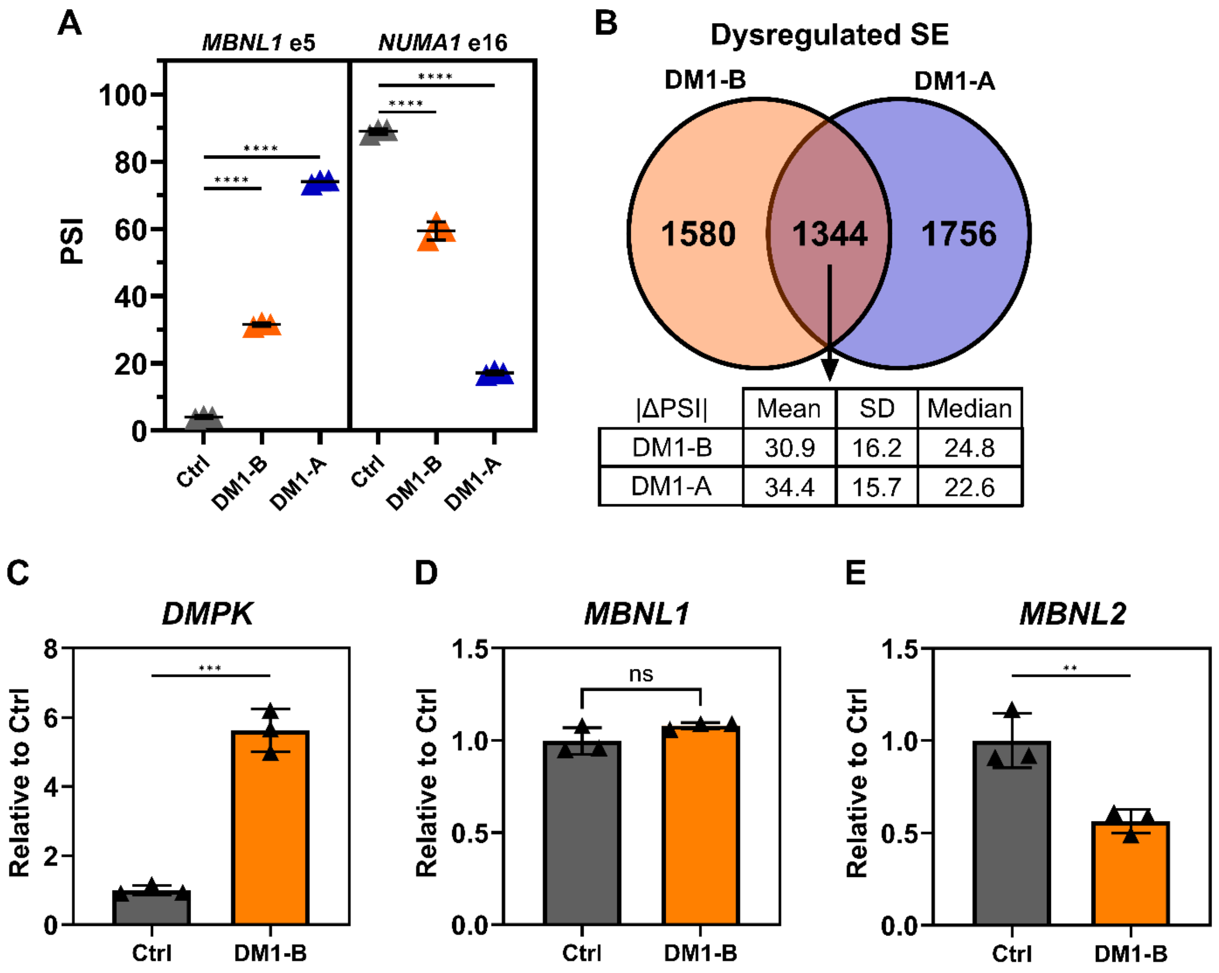

Figure S3. Characterization of the DM1-B myotube cell line. (**A**) RT-PCR analysis of Ctrl (grey), DM1-B (orange), and DM1-A (blue) myotube splicing dysregulation for *MBNL1* exon 5 (left) and *NUMA1* exon 16 (right). (**B**) Venn diagram of dysregulated SE between DM1-B (orange) and DM1-A (blue) myotube models. The mean, standard deviation (SD), and median values for common SE events between DM1-B and DM1-A are noted in the table below. Ordinary one-way ANOVA with Tukey's multiple comparisons test identified significant differences between Ctrl and affected myotube cells (P-adj < 0.05 = *, < 0.01**, < 0.0005***, < 0.0001****). qPCR of relative transcript levels of (**C**) *DMPK*, (**D**) *MBNL1*, and (**E**) *MBNL2* in Ctrl and DM1-B myotubes. Unpaired t-test of gene expression differences between Ctrl and DM1-B groups denote significant differences in *DMPK* (p-value = 0.0002) and *MBNL2* (p-value = 0.0093) expression (p-value < 0.05 = *, < 0.01**, < 0.0005***, ns = non-significant).

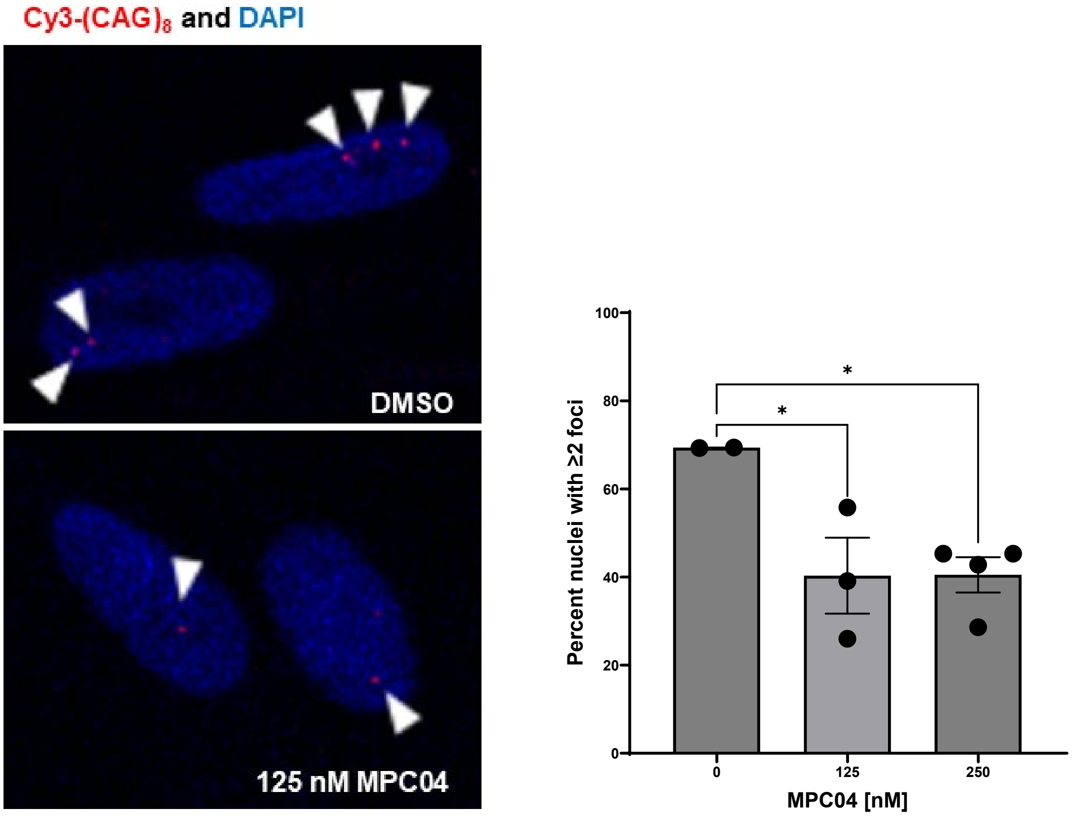

Figure S4. MPC04 treatment reduces CUG ribonuclear foci in DM1-B myotubes. Patient derived myotubes were treated for 96 hours with MPC04. Fluorescence in situ hybridization was performed. Nuclei and foci were quantitated using CellProfiler, the percentage of nuclei with at least 2 foci was calculated for all replicates. P values were determined with one-way Anova and Dunnett's multiple comparisons to the untreated DM1 values. A minimum of 90 nuclei were counted per replicate. (Mean ± SEM) (*P < 0.05).

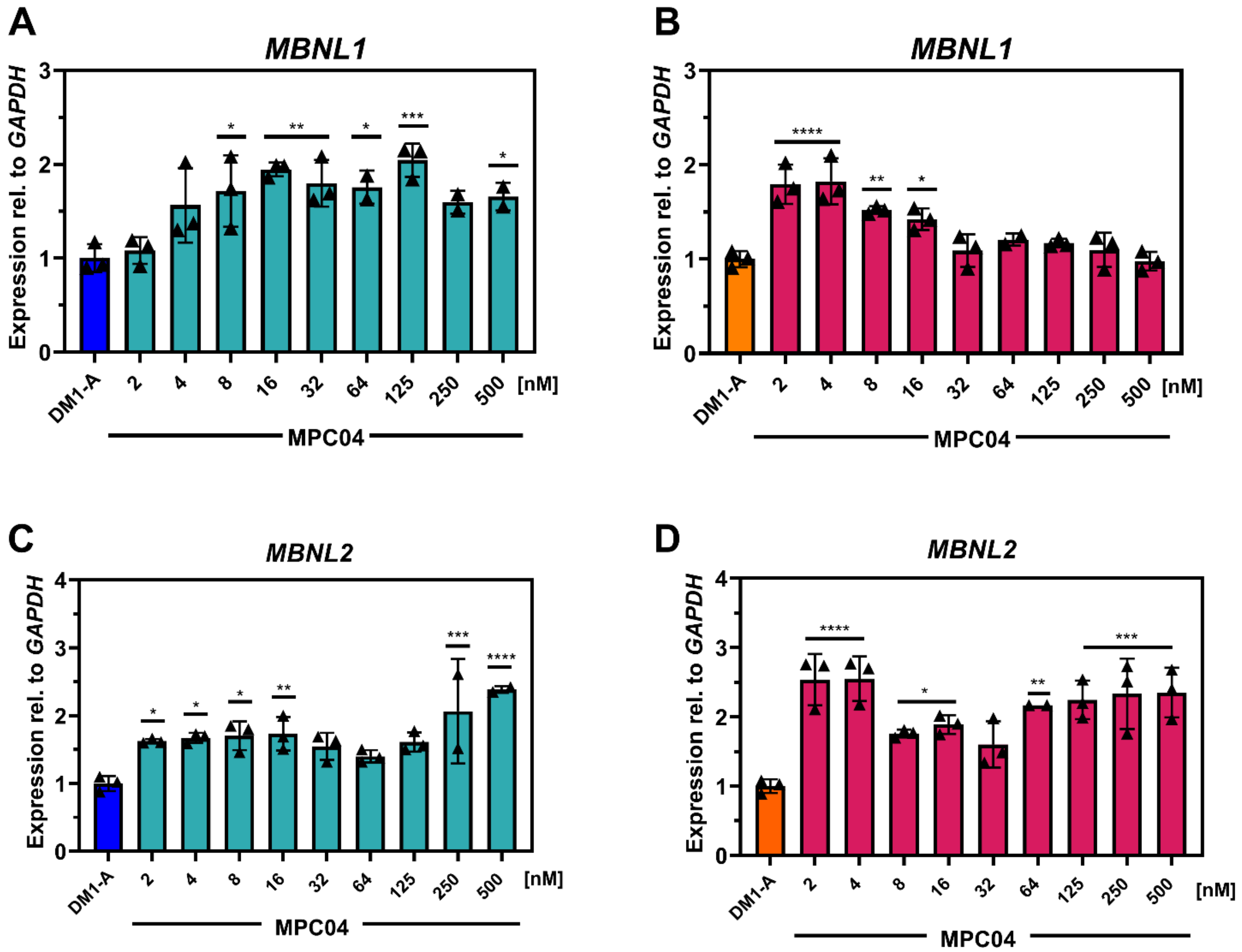

Figure S5. MPC04 treatment increases *MBNL1* and *MBNL2* transcripts in DM1 myotubes. Patient derived myotubes were treated for 96 hours with MPC04. RT-qPCR analysis shows an increase of *MBNL1* and *MBNL2* transcript expression (normalized to *GAPDH* and relative to DMSO treated) in (**A&C**) DM1-A and (**B&D**) DM1-B patient derived myotubes respectively. Adjusted-P values were determined with one-way Anova and Dunnett's multiple comparisons to the untreated DM1 values (n=3 for all treatments, Mean± SEM) (P-adj < 0.05 = *, < 0.01**, < 0.0005***, < 0.0001****).

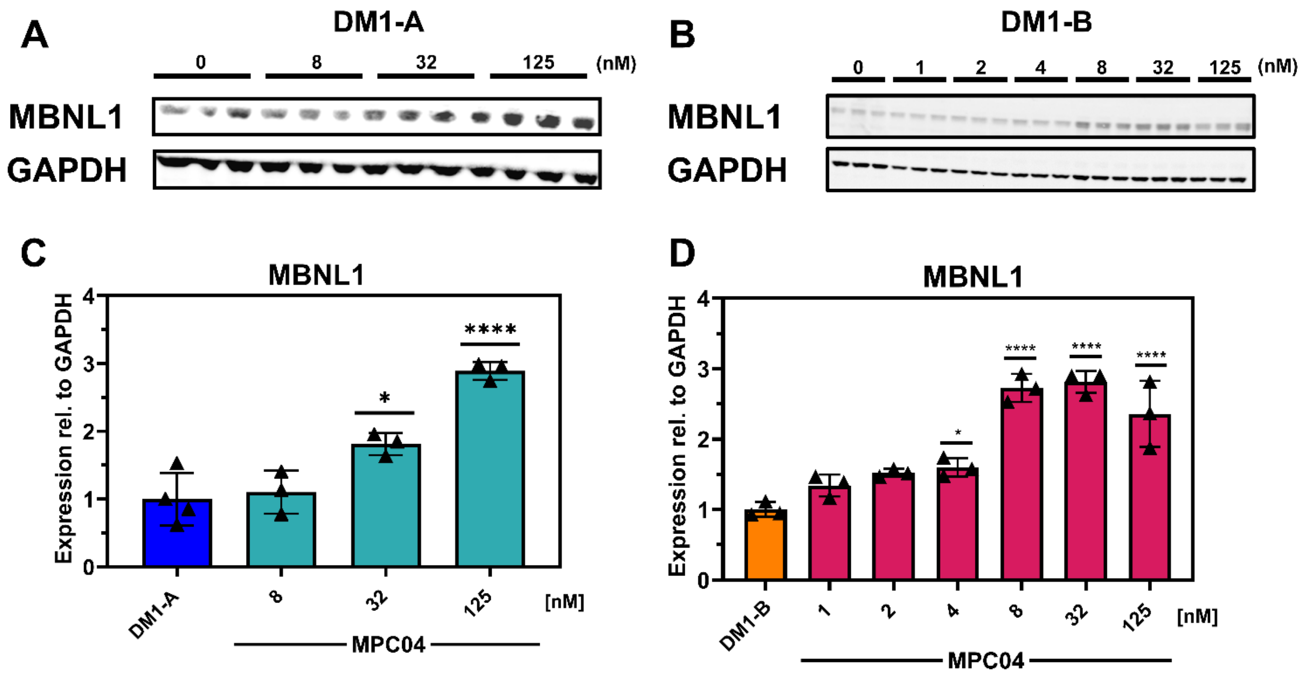

Figure S6. MPC04 treatment affects MBNL1 protein expression in DM1 myotubes Patient derived myotubes were treated with various concentrations of MPC04 for 96 hours. Western blotting for MBNL1 and GAPDH (**A&B**) and quantification (**C&D**) showed a relative MBNL1 protein level increase in (**A&C**) DM1-A and (**B&D**) DM1-B with MPC04 treatment. Adjusted-P values were determined with one-way Anova and Dunnett's multiple comparisons to the untreated DM1 values (n=3 for all treatments, Mean± SEM) (P-adj < 0.05 = *, < 0.01**, < 0.0005***, < 0.0001****).

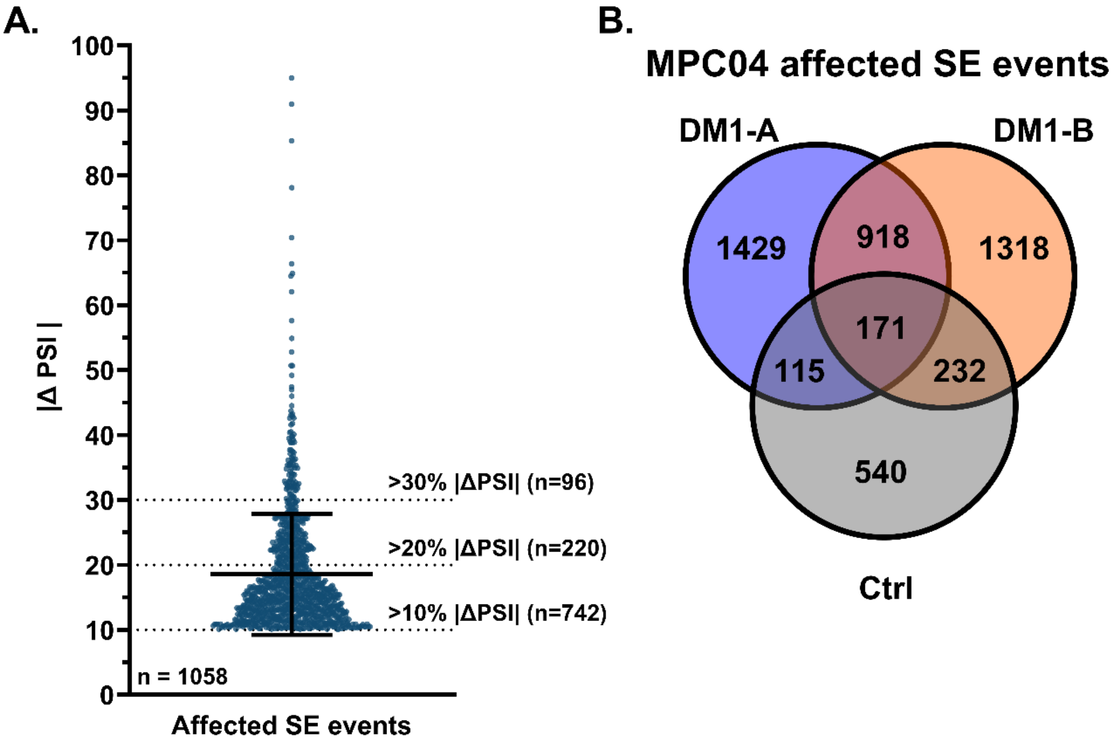

Figure S7. Splicing change following MPC treatment of unaffected control myotubes (**A**) Violin plot of |ΔPSI| of SE events affected by 125nM MPC04 treatment in Ctrl myotubes. Mean and standard deviation are plotted as overlayed black whiskers. Hashed lines denote 10, 20, and 30 |ΔPSI| on the y-axis. The number of events surpassing the indicated hashed values is noted on the right side of the plot. (**B**) Venn diagram of affected SE events in Ctrl myotubes treated with 125 nM MPC04 (grey) and dysregulated SE found in DM1-A (blue) and DM1-B (orange) myotube cell lines.

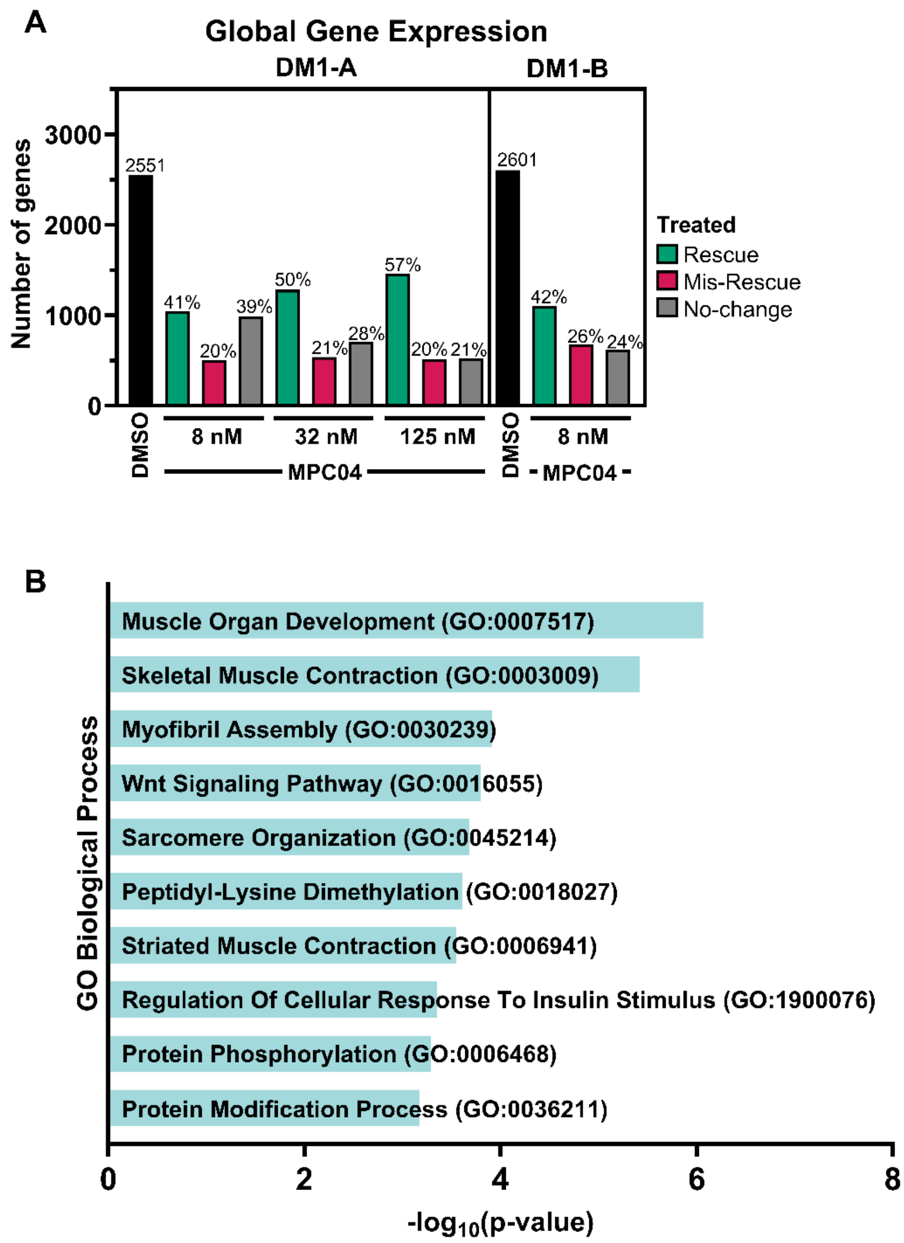

Figure S8. Gene expression effects from MPC04 treatment. (**A**) Global gene expression effected by MPC04 treatment. The number of genes (y-axis) are depicted and grouped by cell type (DM1-A, DM1-B) and condition (dysregulated, DMSO treated): black; ‘Rescue’: green; ‘Mis-rescue’: red; ‘No-change’: grey). The total of dysregulated genes labels the ‘NT’ groups and the percentage of those genes that were categorized as ‘rescue’, ‘mis-rescue’, and ‘no-change’ are shown above each ‘Treated’ result. (**B**) Gene ontology (GO) biological processes (x-axis) and the associated p-values on a log scale (y-axis) depict processes affected by ‘rescue’ categorized genes.

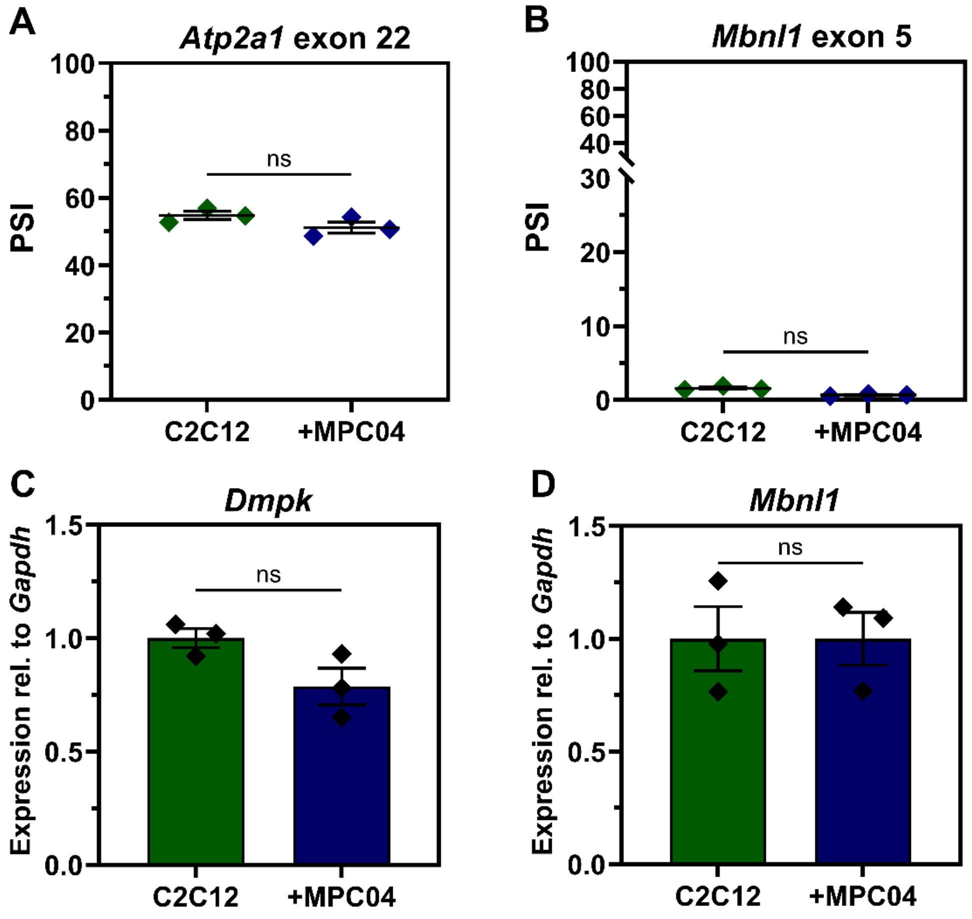

Figure S9. MPC04 does not affect splicing or gene expression in C2C12 mouse myoblasts. Splicing isoform analysis of (**A**) *Atp2a1* exon22 and (**B**) *Mbnl1* exon 5 after treatment with 125 nM MPC04 for 96-hours in C2C12 mouse myoblast cells are plotted. Relative expression of (**C**) *Dmpk* and (**D**) *Mbnl1* to *Gapdh* are plotted and normalized to the untreated C2C12 group. The nonparametric Wilcoxon–Mann–Whitney test was used to compare significant differences between groups (ns = non-significant).

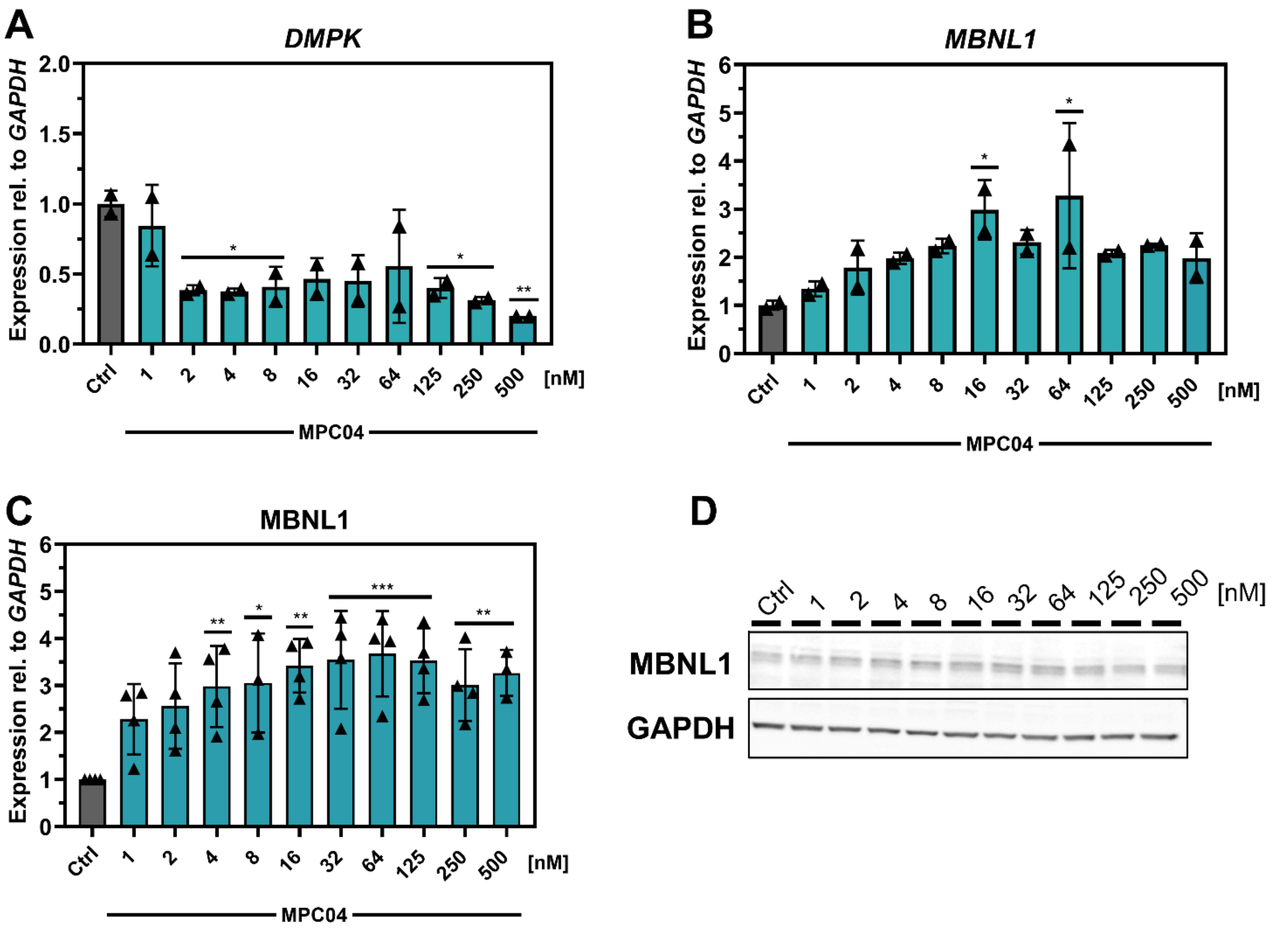

Figure S10. MPC04 reduces *DMPK* transcript levels and increases both *MBNL1* transcript and MBNL1 protein levels in unaffected control myotubes. Control myotubes were treated with multiple concentrations of MPC04 for 96 hours. RNA and protein were extracted from these cells for RT-qPCR and Western blot analysis. MPC04 reduced relative (**A**) *DMPK* transcript levels and increased relative (**B**) *MBNL1* transcript levels in control myotubes. (**C & D**) MBNL1 protein levels were also increased with MPC04 treatment. Adjusted-P values were determined with one-way Anova and Dunnett's multiple comparisons to the untreated unaffected control values (n=4 for all treatments, Mean± SEM (P-adj < 0.05 = *, < 0.01**, < 0.0005***, < 0.0001****).

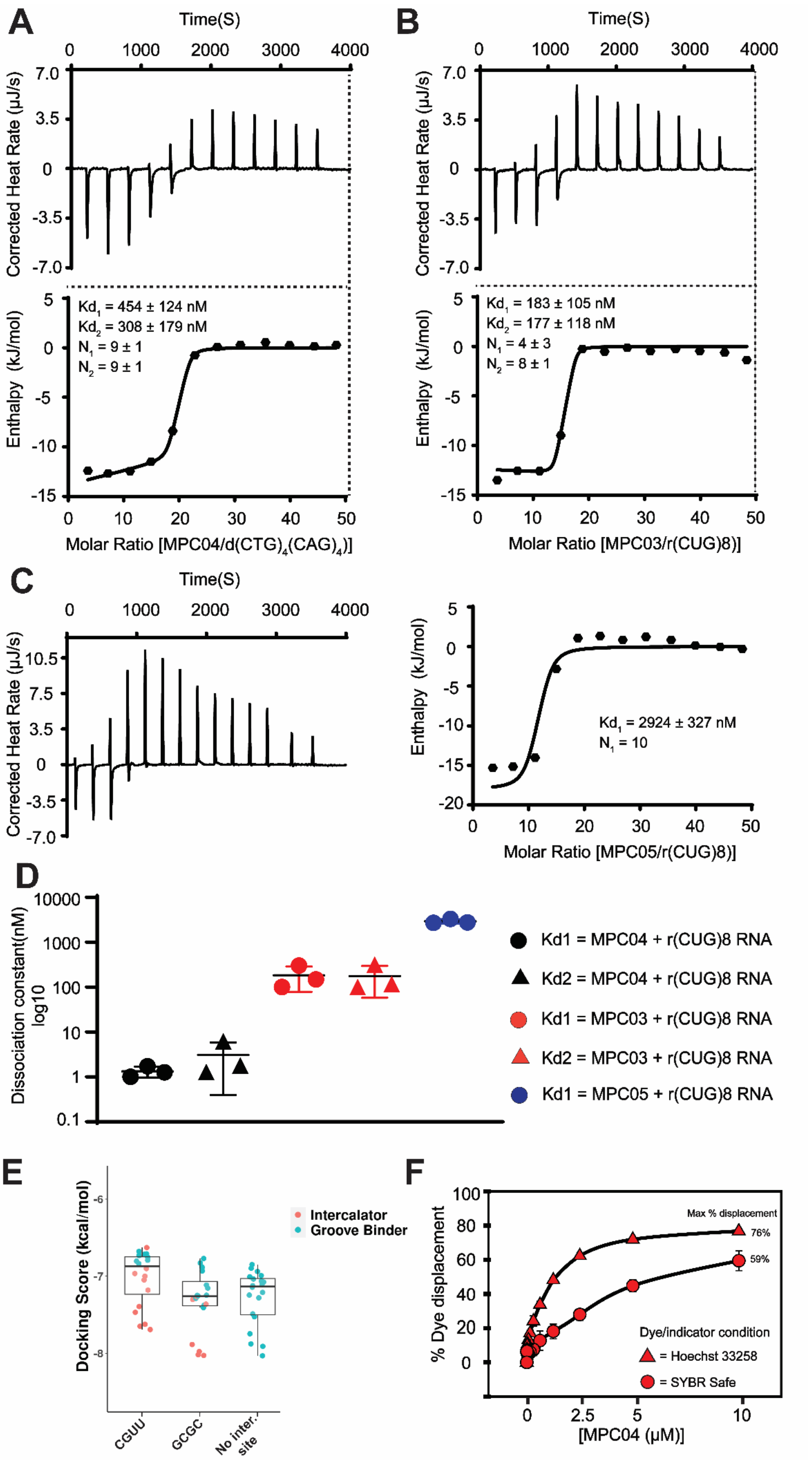
A
Time(S)
Corrected Heat Rate (µJ/s)
Enthalpy (kJ/mol)
Kd1 = 454 ± 124 nM
Kd2 = 308 ± 179 nM
N1 = 9 ± 1
N2 = 9 ± 1
Molar Ratio [MPC04/d(CTG)4(CAG)4)]
B
Kd1 = 183 ± 105 nM
Kd2 = 177 ± 118 nM
N1 = 4 ± 3
N2 = 8 ± 1
Molar Ratio [MPC03/r(CUG)8)]
C
Kd1 = 2924 ± 327 nM
N1 = 10
Molar Ratio [MPC05/r(CUG)8)]
D
Dissociation constant(nM) log10
Kd1 = MPC04 + r(CUG)8 RNA
Kd2 = MPC04 + r(CUG)8 RNA
Kd1 = MPC03 + r(CUG)8 RNA
Kd2 = MPC03 + r(CUG)8 RNA
Kd1 = MPC05 + r(CUG)8 RNA
E
Docking Score (kcal/mol)
Intercalator
Groove Binder
CGUU
GCGC
No inter. site
F
% Dye displacement
Max % displacement
76%
59%
Dye/indicator condition
= Hoechst 33258
= SYBR Safe
[MPC04 (µM)]

Figure S11: ITC analysis of MPC analogues and experimental support for multiple binding modes. **(A–C)** Representative isothermal titration calorimetry (ITC) thermograms and binding isotherms for **(A)** MPC04 with d(CTG)$_4$(CAG)$_4$ DNA, **(B)** MPC03 with r(CUG)$_8$ RNA, and **(C)** MPC05 with r(CUG)$_8$ RNA. The upper panels show the corrected heat rate as a function of time, and the lower panels show the integrated heat change per injection with the corresponding fits. MPC03 binding is best fitted by a multi-site binding model, whereas MPC05 binding is fitted using a single-site model. **(D)** Comparison of ITC-derived dissociation constants (Kd) for MPC04, MPC03, and MPC05 binding to r(CUG)$_8$ RNA, high-lighting the rank-order relationship in RNA binding affinity across the compound series. **(E)** Distribution of docking scores for groove-associated and intercalation-compatible binding poses of MPC04 at different trinucleotide steps of the r(CUG)$_8$ duplex (CGUU constrained, GCGC constrained & no intercalation (inter.) site on the receptor), illustrating that both interaction modes are energetically feasible. **(F)** Fluo-rescence indicator displacement assay showing displacement of Hoechst 33258 (groove-binding indica-tor) and SYBR Safe (intercalation-sensitive dye) from r(CUG)$_8$ RNA by MPC04. Data are plotted as percentage dye displacement as a function of MPC04 concentration.
